# Trends in estrogen replacement therapy use among younger gynecologic cancer survivors, 2013-2021

**DOI:** 10.1016/j.gore.2026.102178

**Published:** 2026-08-05

**Authors:** Christine Hsu Wang, Yong Shan, Gwyn Richardson, Fangjian Guo, Victor Adekanmbi, Thao N. Hoang, Abbey B. Berenson

**Affiliations:** a Department of Obstetrics & Gynecology, University of Texas Medical Branch at Galveston, Galveston, TX, USA; b Center for Interdisciplinary Research in Women's Health, University of Texas Medical Branch at Galveston, Galveston, TX, USA; c Department of Biostatistics & Data Science, University of Texas Medical Branch at Galveston, Galveston, TX, USA; d Department of Gynecologic Oncology and Reproductive Medicine, University of Texas MD Anderson Cancer Center, Houston, TX, USA

**Keywords:** Gynecologic cancer, Estrogen replacement therapy, Premature menopause

## Abstract

**Objectives:**

To evaluate temporal trends in estrogen replacement therapy (ERT) utilization among younger gynecologic cancer survivors.

**Methods:**

We conducted a cross-sectional study using the Merative MarketScan Research Database from January 1, 2013, through December 31, 2021. We included female patients aged 18–51 years with a diagnosis or history of gynecologic cancer. Annual prevalence of ERT use was calculated, and trends over time were assessed using annual percent change (APC) and 95% confidence intervals (CIs). Results were assessed across cancer types and age groups.

**Results:**

ERT utilization increased from 10.9% in 2013 to 14.1% in 2021 among all gynecologic cancer survivors (APC 3.41%, 95% CI 2.13–4.71%). Increasing ERT use was observed across all gynecologic cancer subgroups and age categories during the study period. Ovarian cancer survivors consistently had the greatest prevalence of ERT use, whereas uterine cancer survivors had the lowest prevalence across the study period. Among the age sub-groups, the 18–34 years old age group had the largest increase in ERT utilization, from 6.8% to 10.5% (APC 5.68%, 95% CI 3.1–8.33) over the study period.

**Conclusions:**

ERT utilization among younger gynecologic cancer survivors increased between 2013 and 2021. As ERT use continues to rise in this population, further research is needed to identify patient-, provider-, and system-level factors associated with prescribing and uptake and to evaluate gaps in menopausal survivorship care.

## Highlights

• ERT utilization increased among younger gynecologic cancer survivors from 2013 to 2021.
• The prevalence of ERT increased from 10.9% in 2013 to 14.1% in 2021.
• ERT utilization increased at an average annual percent change of 3.35%.
• ERT utilization increased across all gynecologic cancer types and age sub-groups.
• These findings support the importance of future research in improving evidence-based menopausal care for patients with a history of a gynecologic cancer.

## 1 Background

Cancer survivors experiencing treatment-related menopause often experience worse menopause symptoms compared to women entering menopause naturally, and patients' quality of life can be significantly affected by genitourinary and vasomotor symptoms of menopause (Hickey et al., 2024). The Society of Gynecologic Oncology clinical practice statement, endorsed by the North American Menopause Society, suggests that estrogen replacement therapy (ERT) is considered safe for use in survivors of cancers that are not hormonally-driven (Hickey et al., 2024; Sinno et al., 2020). However, ERT use remains low in this population, likely due to hesitancies around cancer recurrence (Anderson et al., 2004; Cagnacci and Venier, 2019).

In the general population in the United States, ERT utilization dropped precipitously after findings from the 2002 Women's Health Initiative study showed greater risks than benefits with ERT use (Cagnacci and Venier, 2019; Tsai et al., 2011; Commissioner O of the. HHS, 2025). Recent reports suggest renewed interest in the use of ERT in the general U.S. population, as studies support its safe use in selected populations of women (Research T, 2026; *Hormone Replacement Therapy Market Insights* 2026-2036, n.d.). Nevertheless, little is known about trends in the use of ERT among younger gynecologic cancer survivors after cancer treatment in the United States. To our knowledge, only one recent study conducted using data from the United States examined trends in ERT after uterine cancer (Suzuki et al., 2024). The authors found that ERT utilization declined from 2008 to 2020 (Suzuki et al., 2024).

The purpose of the study is, therefore, to describe trends of ERT use in younger gynecologic cancer survivors (Hickey et al., 2024). Specifically, this study examined ERT utilization in survivors of gynecologic cancers treated with pelvic radiation, chemotherapy, or bilateral oophorectomy (Hickey et al., 2024).

## 2 Methods

*Data.* In this cross-sectional study, we used data from the Merative MarketScan Research Database, from January 1, 2012, to December 31, 2021. MarketScan data includes commercially-insured patients in the United States and includes inpatient, outpatient, and prescription drug claims data, as well as eligibility information (Merative, 2022).

*Study population.* For each year from 2013 through 2021, we included patients with an ICD-9 or ICD-10 code for a gynecologic cancer or cancer history between January 1st and December 31st of that year (Supplementary Table 1, Supplementary Table 3). The date for the first diagnosis code in that period was referred to as the “diagnosis date”. Only patients between 18 and 51 years old in the year of interest were included, since we aimed to identify younger women before the average of menopause in the United States (51 years) (Wegrzynowicz et al., 2025). Age was defined using the mid-year age, using the age on July 1st of the year of interest.

Patients were required to have continuous enrollment in the year of interest and in the six months before and after the diagnosis date. Individuals who had a code suggesting active treatment in the 6 months before or 6 months after the diagnosis date were excluded. ICD-9-CM, ICD-10-CM codes were used to identify patients with gynecologic cancer or history of gynecologic cancer. ICD-9-CM, ICD-10-CM, and CPT codes were used to identify patients with chemotherapy, immunotherapy, or radiotherapy. Codes suggesting active treatment were derived from Nitecki and colleagues' study (Nitecki et al., 2022), then further refined with input from a gynecologic oncologist (GR). Individuals with a history of breast cancer were not excluded from our study as some breast cancer survivors may be appropriate candidates for estrogen-containing therapies, such as low-dose vaginal estrogen for managing genitourinary symptoms of menopause.

ERT was identified using NDC codes, and we characterized ERT by route of administration and formulation, including oral estradiol, oral conjugated estrogens, oral esterified estrogens, transdermal estrogen, vaginal estrogen, intramuscular estrogen, and combined estrogen-progestogen therapy. For those with claims for more than one ERT formulation, the first ERT prescription claim was used to classify the route of administration and formulation. Diagnoses codes and NDC codes are presented in Supplementary Tables 1 and 2.

### 2.1 Analysis

We estimated the annual prevalence of ERT use for each calendar year (2013−2021), defined as the number of gynecologic cancer survivors with at least one ERT medication in the calendar year of interest divided by the number of gynecologic cancer survivors in that same year. Patients could contribute more than one count of ERT use over separate calendar years if they met the inclusion and exclusion criteria for the specific year. We used weighted log-linear regression models to evaluate trends in ERT use, determining annual percent change (APC) and 95% confidence intervals (CIs). We also assessed results across cancer types and age groups, using generalized estimating equation models with interaction terms between the calendar year and subgroup variables. The study was considered exempt by our institution's IRB, given the use of deidentified data. SAS for Windows version 9.4 was used for the analysis.

## 3 Results

The study cohort included 59,832 gynecologic cancer survivors between the ages of 18–51. The mean age was 41.6 years (std 7.7 years). The three most common types of cancer were cervical cancer (36.0%), ovarian cancer (33.7%), and uterine cancer (21.9%). Among ERT users, oral estradiol was the most common initial formulation identified in claims (30.8%), followed by vaginal estrogen (24.6%), transdermal estrogen (24.6%), oral conjugated estrogens (14.2%), combined estrogen-progestogen therapy (3.5%), oral esterified estrogens (1.8%), and intramuscular estrogen (0.3%). The oral route of administration was the most common (50.3%), followed by vaginal (24.6%), transdermal (23.3%), topical (1.5%), and intramuscular (0.3%) routes.

**Fig. 1:**
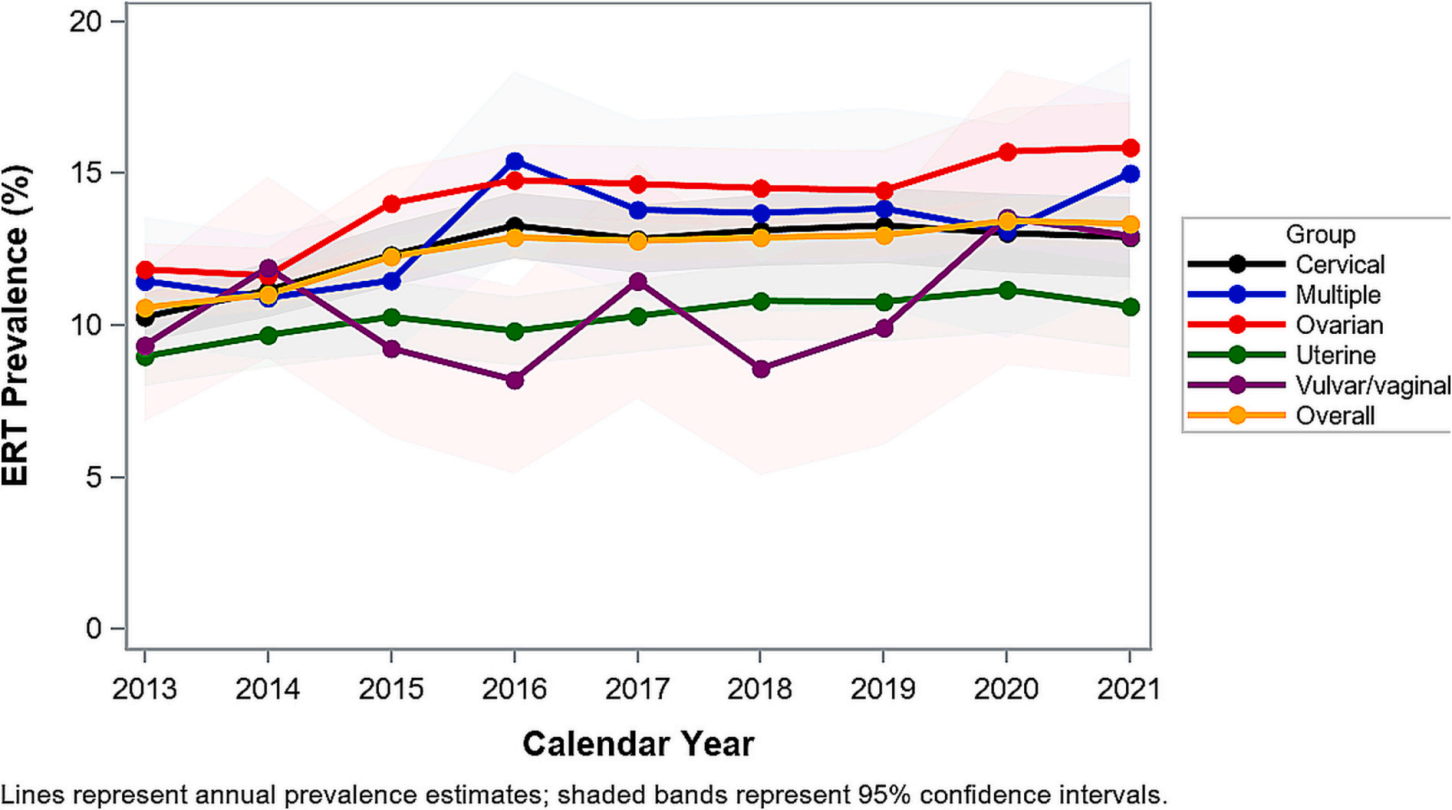
Annual Prevalence of ERT use overall and by cancer type.

Between 2013 and 2021, the prevalence of ERT use among gynecologic cancer survivors increased from 10.9% to 14.1% (Table 1, Fig. 1), corresponding to an APC of 3.41% (95% CI 2.13–4.71%) (Table 2). Though APC varied across cancer types, the overall temporal trends in ERT use did not differ significantly by cancer site (interaction *p* = 0.51). Ovarian cancer survivors had the largest increase in ERT utilization (APC 4.34%, 95% CI 2.41–6.31%). Vulvar/vaginal cancer survivors had no statistically significant change in ERT utilization over time (APC 3.03%, 95% CI −1.78-8.06%) (Table 2, Supplementary Table 3). Across the study period, ovarian cancer survivors consistently had the greatest prevalence of ERT use, whereas uterine cancer survivors had the lowest prevalence.

**Table 1:** Overall annual prevalence of ERT utilization, 2013–2021.

Year	ERT Users	Total Survivors	Prevalence	95% CI
2013	1685	15,529	10.85%	10.36–11.34%
2014	1539	13,734	11.21%	10.68–11.73%
2015	1414	11,293	12.52%	11.91–13.13%
2016	1414	10,731	13.18%	12.54–13.82%
2017	1284	9745	13.18%	12.5–13.85%
2018	1186	8901	13.32%	12.62–14.03%
2019	1117	8333	13.40%	12.67–14.14%
2020	1050	7474	14.05%	13.26–14.84%
2021	1002	7131	14.05%	13.24–14.86%

**Table 2:** Overall prevalence and annual percent change in ERT prevalence, 2013–2021.

Group	Prevalence 2013, % (95% CI)	Prevalence 2021, % (95% CI)	APC (%)	95% CI
Overall	10.6% (10.10–11.06%)	13.3% (12.55–14.13%)	3.41	2.13 to 4.71

Cancer Type				
Vulvar/vaginal	9.52% (7.01–12.03%)	13.93% (9.14–18.72%)	3.03	-1.78 to 8.06
Cervical	10.52% (9.71–11.34%)	13.68% (12.31–15.04%)	3.45	1.65 to 5.27
Uterine	9.16% (8.17–10.15%)	10.88% (9.47–12.29%)	2.43	1.38 to 3.48
Ovarian			4.34	2.41 to 6.31
Multiple	11.45% (9.35–13.55%)	15.59% (11.73–19.44%)	3.97	0.6 to 7.45

Age Group				
18–34 years	6.82% (5.86–7.79%)	10.47% (8.62–12.32%)	5.68	3.1 to 8.33
35–39 years	9.81% (8.58–11.05%)	12.72% (10.8–14.64%)	4.32	2.05 to 6.64
40–44 years	11.29% (10.23–12.35%)	15.60% (13.84–17.36%)	4.19	2.67 to 5.72
45–51 years	12.41% (11.65–13.17%)	14.90% (13.68–16.12%)	2.26	0.72 to 3.82

Average annual percent change estimated via weighted log-linear regression models, 2013–2021.

**Fig. 2:**
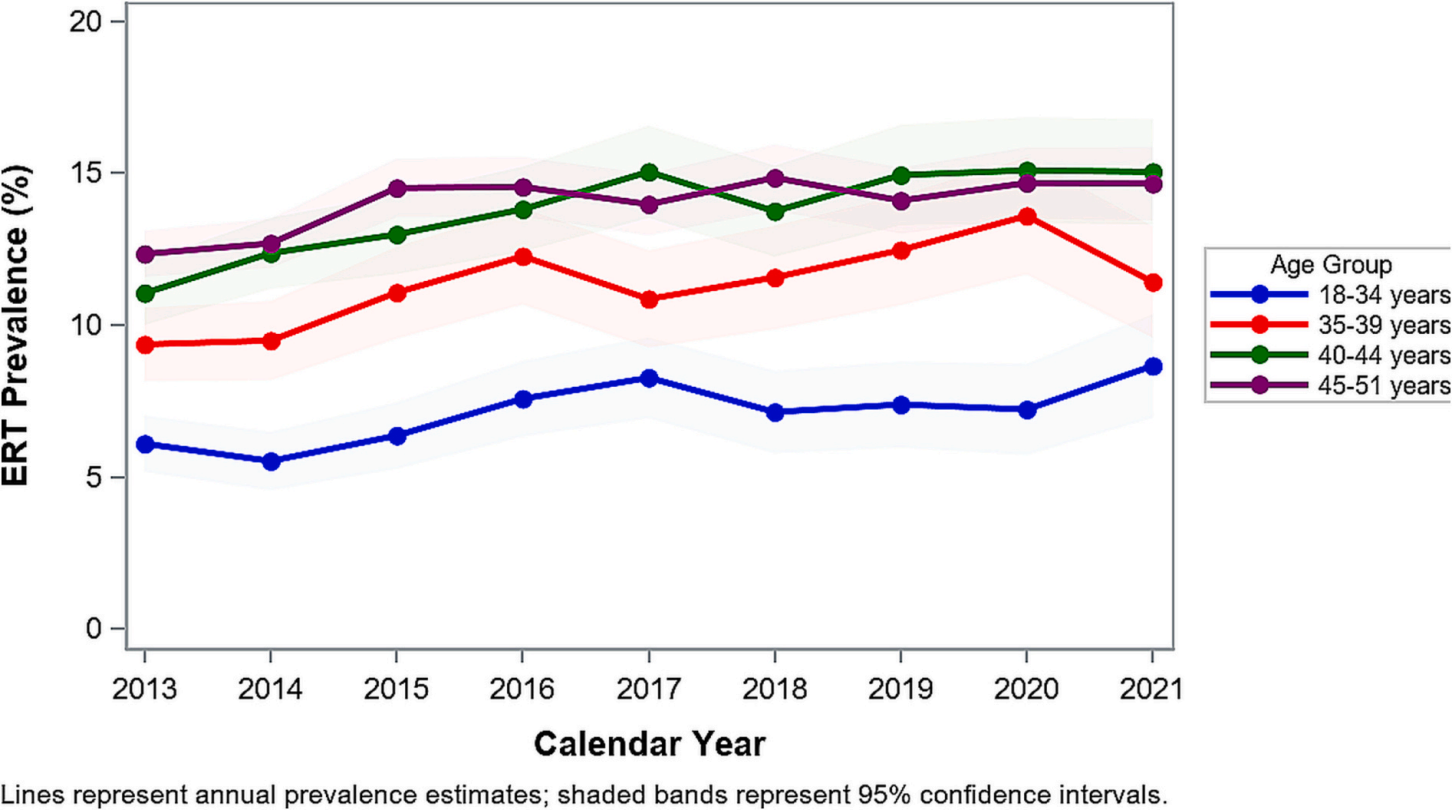
Annual Prevalence of ERT use by age group.

Among the age subgroups, the overall prevalence of ERT use remained highest among those in the 40–44 and 45–51 age groups while ERT prevalence remained low among those 18–34 years old (Fig. 2). Nevertheless, the 18–34 years old age group had the largest increase in ERT utilization (APC 5.68%, 95% CI 3.1–8.33) over the study period. The oldest age group (45–51 years old) had the lowest increase in ERT utilization over time (APC 2.26%, 95% CI 0.72–3.82). Though APC varied across age groups, the overall temporal trends in ERT use did not differ significantly by age group (interaction *p* = 0.052) (Table 2, Supplementary Table 4).

## 4 Discussion

ERT utilization increased significantly between 2013 and 2021 in this nationwide cohort of younger gynecologic cancer survivors. It is likely ERT utilization will continue to increase in this population, especially with the release and implementation of the Society of Gynecologic Oncology's 2020 clinical practice statement, which provides guidance for ERT use in women with gynecologic cancers. The clinical practice statement emphasizes that estrogen therapy is considered safe in the following groups: (1) women with early-stage endometrial cancer; (2) most women with epithelial ovarian cancers, except those with hormone sensitive subtypes; and (3) women with cervical cancer with induced menopause (Sinno et al., 2020). It is important to recognize, however, that clinicians and patients may remain hesitant towards ERT use after a gynecologic cancer diagnosis (Gorman and Shih, 2025; Halldorsdottir et al., 2018), and translation efforts are integral for implementing guideline recommendations into routine clinical practice.

Additionally, we found that ERT use differed across cancer types, as expected. These differences likely reflect variation in perceived risk and clinician comfort with ERT prescribing, especially with greater concerns with estrogen use among endometrial cancer survivors. Age-related differences were also observed, with higher utilization among survivors aged 40–44 and 45–51 years but the greatest APC among survivors aged 18–34 years. The increase in ERT utilization among younger survivors may reflect both the greater burden of treatment-induced menopause symptoms and the growing recognition of the long-term consequences of premature menopause, including cardiovascular disease risk, osteoporosis risk, vasomotor symptoms, sexual dysfunction, and more (Faubion et al., 2015).

The findings from this study should be considered in light of several limitations. First, not all gynecologic cancer survivors are appropriate candidates for ERT or require treatment with ERT, though these nuances are not well-captured using claims data. Menopausal status and treatment appropriateness could not be determined from claims data because menopause symptoms and ovarian function are not reliably captured in administrative claims data. Therefore, our findings reflect patterns of ERT utilization rather than prevalence of menopause or unmet treatment need in this population. Although a portion of our cohort had a history of breast cancer (*N* = 2467; 4.1%), these patients were not excluded because the study aimed to describe overall estrogen-containing therapy utilization among gynecologic cancer survivors. However, a history of breast cancer may substantially influence hormone therapy decision-making and may contribute to observed differences in ERT utilization.

Another limitation is that claims-based medication data reflects filling of the prescription abut lacks insight on whether the patient ultimately used the medication. The MarketScan data also only includes a commercially insured population and is not generalizable to those with Medicaid, Medicare, or no insurance. Furthermore, information regarding patient preferences, clinical decision-making, cancer stage and subtype, and menopausal symptom frequency and severity were not available, limiting our ability to determine ERT appropriateness. Lastly, the study assessed annual prevalence of ERT use and does not assess treatment adherence over time. Our study also has several strengths. To our knowledge, this is the first study using nationwide data assessing temporal trends in ERT use in younger gynecologic cancer survivors. The use of large, administrative claims data allows a large sample size for evaluating ERT use across multiple gynecologic cancer types, allowing subgroup analysis by cancer type and age group. The study also spans 2013 to 2021, providing baseline trend data on ERT use before and after the SGO 2020 clinical practice statement.

In conclusion, ERT utilization increased between 2013 and 2021 across all gynecologic cancers and age groups included in the study. However, overall use of ERT among gynecologic cancer survivors remained relatively low. It is important to note, though, that these findings should not be interpreted as evidence of unmet need, as clinical factors, such as menopausal status, ovarian function, symptom burden, and contraindications, were not available in the data source. Although non-hormonal therapies for vasomotor symptoms, such as fezolinetant, have emerged in recent years, their long-term safety and potential cancer-related risk continue to be evaluated (Boretti et al., 2026; Lederman et al., 2023). ERT therefore remains an important treatment option for appropriately selected gynecologic cancer survivors experiencing premature menopause, with the potential to improve quality of life in survivorship care. As ERT use continues to increase among gynecologic cancer survivors, further research is needed to identify patient-, provider-, and health system-level determinants of prescribing and uptake and to characterize gaps in menopausal survivorship care.

## Declaration of generative AI and AI-assisted technologies in the manuscript preparation process

During the preparation of this work, the authors used ChatGPT (OpenAI) to assist with analytic programming support (refinement of the analytic code and troubleshooting of coding issues) and language editing in preparing the manuscript. All code generated and modified with AI assistance was reviewed and validated by the authors. The authors retain full responsibility for the study design, cohort definition, statistical analyses, and interpretation of findings. The authors take full responsibility for the content of the published article.

## CRediT authorship contribution statement

**Christine Hsu Wang:** Writing – review & editing, Writing – original draft, Project administration, Methodology, Formal analysis, Data curation, Conceptualization. **Yong Shan:** Writing – review & editing, Formal analysis, Data curation. **Gwyn Richardson:** Writing – review & editing, Conceptualization. **Fangjian Guo:** Writing – review & editing, Conceptualization. **Victor Adekanmbi:** Writing – review & editing, Conceptualization. **Thao N. Hoang:** Writing – review & editing, Conceptualization. **Abbey B. Berenson:** Writing – review & editing, Supervision, Funding acquisition, Conceptualization.

## Funding

Drs. Hsu and Adekanmbi are supported by a research career development award (K12AR084228: Building Interdisciplinary Research Careers in Women's Health Program-BIRCWH; Berenson, PI) from the National Institutes of Health/Office of the Director (OD) and National Institute of Arthritis and Musculoskeletal and Skin Disease (NIAMS). The content is solely the responsibility of the authors and does not necessarily represent the official views of the National Institutes of Health.

## Declaration of competing interest

The authors have no conflicts of interest to disclose.

The content is solely the responsibility of the authors and does not necessarily represent the official views of the National Institutes of Health.

## Appendix A Supplementary data

Supplementary material 1 Supplementary Table 1 lists the specific ICD-9 and ICD-10 codes applied to Marketscan data to identify eligible patients and remove ineligible cases.

Supplementary material 2 Supplementary Table 2 includes National Drug Codes for identifying ERT. Supplementary Table 3 details the steps for the cohort definition and cohort counts per year, using 2013 as an example. Supplementary Table 4 shows the annual prevalence of ERT use by cancer type. Supplementary Table 5 shows the annual prevalence of ERT use by age groups.
