# Supplementary material for: Trends in estrogen replacement therapy use among younger gynecologic cancer survivors, 2013-2021
Source: Gynecol Oncol Rep. 2026 Aug 5;66:102178. doi: 10.1016/j.gore.2026.102178 (PMC13476577; doi:10.1016/j.gore.2026.102178)
Supplement: Supplementary file 1 [file mmc1.docx]

**Supplementary Table 1. ICD-9, ICD-10, and CPT Codes for Inclusion and Exclusion Criteria.**

*Inclusion criteria codes*

| **Condition** | **ICD-9-CM** | **ICD-10-CM** | **Description** |
| --- | --- | --- | --- |
| Vulvar cancer | 184.1, 184.2, 184.3, 184.4 | C51.x | Malignant neoplasm of vulva |
| Vaginal cancer | 184.0 | C52 | Malignant neoplasm of vagina |
| Cervical cancer | 180.0, 180.1, 180.8, 180.9 | C53.x | Malignant neoplasm of cervix uteri |
| Cervical cancer history | V10.41 | Z85.41 | Personal history of malignant neoplasm of cervix uteri |
| Uterine cancer | 182.0, 182.1 | C54.x | Malignant neoplasm of corpus uteri |
| Uterine cancer | 182.8 | C55.x | Malignant neoplasm of uterus, part unspecified |
| Uterine cancer history | V10.42 | Z85.42 | Personal history of malignant neoplasm of other parts of uterus |
| Ovarian cancer | 183.0 | C56.x | Malignant neoplasm of ovary |
| Ovarian cancer (Fallopian tube cancer) | 183.2, 183.3, 183.4, 183.5, 183.8, 183.9, 184.8, 184.9 | C57.x | Malignant neoplasm of other and unspecified female genital organs |
| Ovarian cancer (Primary peritoneal cancer) | 158.8, 158.9 | C48.1, C48.2, C48.8 | Malignant neoplasm of specified parts of peritoneum |
| Ovarian cancer history | V10.43 | Z85.43 | Personal history of malignant neoplasm of ovary |

*Codes used to define active treatment*

| **Procedure** | **ICD-9** | **CPT** | **ICD-10** |
| --- | --- | --- | --- |
| Brachytherapy | 92.27 | 57156, 55920, 77326, 77327, 77328, 77750, 77761-77789 | D717, DU12, D7179, D71797, D71797Z, D71798, D71798Z, D71799, D71799Z, D7179B, D7179BZ , D7179C, D7179CZ, D7179Y, D7179YZ, D717B, D717B7, D717B7Z, D717B8, D717B8Z, D717B9, D717B9Z, D717BB, D717BB1, D717BBZ, D717BC, D717BCZ , D717BY, D717BYZ, DU1297Z, DU1298Z, DU1299Z, DU129BZ, DU129CZ, DU129YZ, DU12B7Z, DU12B8Z, DU12B9Z DU12BB1, DU12BBZ, DU12BCZ, DU12BYZ |
| External beam radiation | 92.2, 92.20-92.27, 92.29, 92.3, 92.30-92.39, 92.4, 92.41 | 77371-77373, 77401-77416, 77418, 77422-77425, 77520-77525, 77761-77789, G0174, G0251, G0339, G0340, 0182T | DU020ZZ , DU021ZZ, DU022ZZ, DU023Z0, DU023ZZ, DU024ZZ, DU025ZZ, DU026ZZ DWY6 |
| Chemotherapy | V581, V5811, V5812, V662, V672, 9925, 9928 | J9045, J9060, J9062, C9418, J9000, J9001, J9002, Q2048, Q2049, Q2050, J9190, J9201, C9127, C9431, J9264, J9265, J9267, 96400, 96401, 96402, 96405, 96406, 96408, 96409, 96410, 96411, 96412, 96413, 96414, 96415, 96416, 96417, 96420, 96422, 96423, 96425, 96440, 96445, 96446, 96450, 96500, 96501, 96504, 96505, 96508, 96509, 96510, 96511, 96512, 96520, 96521, 96522, 96523, 96524, 96526, 96530, 96535, 96538, 96540, 96542, 96545, 96549, C8953, C8954, C8955, G0355, G0356, G0357, G0358, G0359, G0360, G0361, G0362, Q0083, Q0084, Q0085, S9329, S9330, S9331, S9338, 99555, J8999, J9999 | Z51.11, 3E00X05, 3E03305, 0JH63VZ |
|  |  |  |  |
| *Also excluded patients with cervical cancer and presence of any of these codes suggesting active treatment:* |  |  |  |
| Radiation |  | 77401-77425 | DWY3, DWY6 |
|  |  |  |  |
| *Also excluded patients with ovarian cancer and presence of any of these codes suggesting active treatment:* |  |  |  |
| Chemo (IP) |  | 96446 |  |

**Supplementary Table 3. Steps for Cohort Definition and Cohort Counts Per Year.**

Using 2013 as an example, these are the steps used for defining the cohort:

| **Step 1** | Include if patient has a code for gynecologic cancer or cancer history between 1/1/2013-12/31/2013. The date for the first diagnosis code between 1/1/2013-12/31/2013 will be referred to as the "diagnosis date". |
| --- | --- |
| **Step 2** | Include females who are 18-51 years old between 1/1/2013-12/31/2013. |
| **Step 3** | Include if patient has continuous enrollment between 1/1/2013-12/31/2013 |
| **Step 4** | Include if patient has continuous enrollment in the six months before and after the diagnosis date. |
| **Step 5** | Exclude if patient has any code for active treatment in the 6 months before or 6 months after the diagnosis date. |

| **Year** | **Total included in cohort** | **Cancer type** | | | | **Total enrolled in MarketScan** |
| --- | --- | --- | --- | --- | --- | --- |
|  |  | Vulvar or Vaginal | Cervical | Uterine | Ovarian |  |
| 2013 | 15,269 | 568 | 5828 | 3943 | 5791 | 49184746 |
| 2014 | 13,924 | 548 | 5385 | 3800 | 5343 | 51440471 |
| 2015 | 11,597 | 447 | 4437 | 3261 | 4313 | 37078988 |
| 2016 | 11,063 | 372 | 4155 | 3334 | 3999 | 35567240 |
| 2017 | 10,217 | 340 | 3854 | 3131 | 3645 | 31291481 |
| 2018 | 9,389 | 307 | 3563 | 2836 | 3342 | 28928765 |
| 2019 | 8,723 | 285 | 3211 | 2653 | 3143 | 26942865 |
| 2020 | 7,808 | 251 | 2844 | 2399 | 2817 | 24929462 |
| 2021 | 7,466 | 245 | 2760 | 2345 | 2615 | 24150915 |

**Supplementary Table 4. Annual Prevalence of ERT Use by Cancer Type.**

| **Year** | **Cancer Type** | **ERT Users** | **Total Survivors** | **Prevalence** | **95% CI** |
| --- | --- | --- | --- | --- | --- |
| 2013 | Cervical | 570 | 5416 | 10.52% | 9.71-11.34% |
| 2014 | Cervical | 542 | 4770 | 11.36% | 10.46-12.26% |
| 2015 | Cervical | 501 | 3981 | 12.58% | 11.55-13.62% |
| 2016 | Cervical | 508 | 3759 | 13.51% | 12.42-14.61% |
| 2017 | Cervical | 450 | 3420 | 13.16% | 12.02-14.29% |
| 2018 | Cervical | 432 | 3170 | 13.63% | 12.43-14.82% |
| 2019 | Cervical | 395 | 2876 | 13.73% | 12.48-14.99% |
| 2020 | Cervical | 352 | 2578 | 13.65% | 12.33-14.98% |
| 2021 | Cervical | 333 | 2435 | 13.68% | 12.31-15.04% |
| 2013 | Multiple | 101 | 882 | 11.45% | 9.35-13.55% |
| 2014 | Multiple | 99 | 899 | 11.01% | 8.97-13.06% |
| 2015 | Multiple | 75 | 654 | 11.47% | 9.03-13.91% |
| 2016 | Multiple | 93 | 584 | 15.92% | 12.96-18.89% |
| 2017 | Multiple | 74 | 529 | 13.99% | 11.03-16.94% |
| 2018 | Multiple | 61 | 431 | 14.15% | 10.86-17.44% |
| 2019 | Multiple | 59 | 419 | 14.08% | 10.75-17.41% |
| 2020 | Multiple | 46 | 351 | 13.11% | 9.58-16.64% |
| 2021 | Multiple | 53 | 340 | 15.59% | 11.73-19.44% |
| 2013 | Ovarian | 665 | 5441 | 12.22% | 11.35-13.09% |
| 2014 | Ovarian | 558 | 4685 | 11.91% | 10.98-12.84% |
| 2015 | Ovarian | 536 | 3719 | 14.41% | 13.28-15.54% |
| 2016 | Ovarian | 529 | 3472 | 15.24% | 14.04-16.43% |
| 2017 | Ovarian | 472 | 3058 | 15.43% | 14.15-16.72% |
| 2018 | Ovarian | 425 | 2832 | 15.01% | 13.69-16.32% |
| 2019 | Ovarian | 411 | 2708 | 15.18% | 13.83-16.53% |
| 2020 | Ovarian | 403 | 2418 | 16.67% | 15.18-18.15% |
| 2021 | Ovarian | 383 | 2271 | 16.86% | 15.32-18.4% |
| 2013 | Uterine | 299 | 3265 | 9.16% | 8.17-10.15% |
| 2014 | Uterine | 285 | 2926 | 9.74% | 8.67-10.81% |
| 2015 | Uterine | 266 | 2560 | 10.39% | 9.21-11.57% |
| 2016 | Uterine | 259 | 2611 | 9.92% | 8.77-11.07% |
| 2017 | Uterine | 258 | 2476 | 10.42% | 9.22-11.62% |
| 2018 | Uterine | 244 | 2223 | 10.98% | 9.68-12.28% |
| 2019 | Uterine | 227 | 2098 | 10.82% | 9.49-12.15% |
| 2020 | Uterine | 223 | 1935 | 11.52% | 10.1-12.95% |
| 2021 | Uterine | 205 | 1884 | 10.88% | 9.47-12.29% |
| 2013 | Vulvar/vaginal | 50 | 525 | 9.52% | 7.01-12.03% |
| 2014 | Vulvar/vaginal | 55 | 454 | 12.11% | 9.11-15.12% |
| 2015 | Vulvar/vaginal | 36 | 379 | 9.50% | 6.55-12.45% |
| 2016 | Vulvar/vaginal | 25 | 305 | 8.20% | 5.12-11.28% |
| 2017 | Vulvar/vaginal | 30 | 262 | 11.45% | 7.59-15.31% |
| 2018 | Vulvar/vaginal | 24 | 245 | 9.80% | 6.07-13.52% |
| 2019 | Vulvar/vaginal | 25 | 232 | 10.78% | 6.79-14.77% |
| 2020 | Vulvar/vaginal | 26 | 192 | 13.54% | 8.7-18.38% |
| 2021 | Vulvar/vaginal | 28 | 201 | 13.93% | 9.14-18.72% |

**Supplementary Table 5. Annual Prevalence of ERT Use by Age Group.**

| **Year** | **Cancer Type** | **ERT Users** | **Total Survivors** | **Prevalence** | **95% CI** |
| --- | --- | --- | --- | --- | --- |
| 2013 | 18-34 years | 178 | 2609 | 6.82% | 5.86-7.79% |
| 2014 | 18-34 years | 137 | 2209 | 6.20% | 5.2-7.21% |
| 2015 | 18-34 years | 143 | 1949 | 7.34% | 6.18-8.49% |
| 2016 | 18-34 years | 146 | 1769 | 8.25% | 6.97-9.54% |
| 2017 | 18-34 years | 154 | 1657 | 9.29% | 7.9-10.69% |
| 2018 | 18-34 years | 116 | 1401 | 8.28% | 6.84-9.72% |
| 2019 | 18-34 years | 119 | 1327 | 8.97% | 7.43-10.5% |
| 2020 | 18-34 years | 105 | 1163 | 9.03% | 7.38-10.68% |
| 2021 | 18-34 years | 110 | 1051 | 10.47% | 8.62-12.32% |
| 2013 | 35-39 years | 219 | 2232 | 9.81% | 8.58-11.05% |
| 2014 | 35-39 years | 193 | 1960 | 9.85% | 8.53-11.17% |
| 2015 | 35-39 years | 185 | 1662 | 11.13% | 9.62-12.64% |
| 2016 | 35-39 years | 215 | 1655 | 12.99% | 11.37-14.61% |
| 2017 | 35-39 years | 170 | 1455 | 11.68% | 10.03-13.33% |
| 2018 | 35-39 years | 163 | 1357 | 12.01% | 10.28-13.74% |
| 2019 | 35-39 years | 166 | 1283 | 12.94% | 11.1-14.77% |
| 2020 | 35-39 years | 176 | 1221 | 14.41% | 12.44-16.38% |
| 2021 | 35-39 years | 147 | 1156 | 12.72% | 10.8-14.64% |
| 2013 | 40-44 years | 387 | 3429 | 11.29% | 10.23-12.35% |
| 2014 | 40-44 years | 380 | 3056 | 12.43% | 11.26-13.6% |
| 2015 | 40-44 years | 337 | 2549 | 13.22% | 11.91-14.54% |
| 2016 | 40-44 years | 327 | 2331 | 14.03% | 12.62-15.44% |
| 2017 | 40-44 years | 326 | 2140 | 15.23% | 13.71-16.76% |
| 2018 | 40-44 years | 289 | 2022 | 14.29% | 12.77-15.82% |
| 2019 | 40-44 years | 273 | 1794 | 15.22% | 13.56-16.88% |
| 2020 | 40-44 years | 254 | 1623 | 15.65% | 13.88-17.42% |
| 2021 | 40-44 years | 255 | 1635 | 15.60% | 13.84-17.36% |
| 2013 | 45-51 years | 901 | 7259 | 12.41% | 11.65-13.17% |
| 2014 | 45-51 years | 829 | 6509 | 12.74% | 11.93-13.55% |
| 2015 | 45-51 years | 749 | 5133 | 14.59% | 13.63-15.56% |
| 2016 | 45-51 years | 726 | 4976 | 14.59% | 13.61-15.57% |
| 2017 | 45-51 years | 634 | 4493 | 14.11% | 13.09-15.13% |
| 2018 | 45-51 years | 618 | 4121 | 15.00% | 13.91-16.09% |
| 2019 | 45-51 years | 559 | 3929 | 14.23% | 13.14-15.32% |
| 2020 | 45-51 years | 515 | 3467 | 14.85% | 13.67-16.04% |
| 2021 | 45-51 years | 490 | 3289 | 14.90% | 13.68-16.12% |
