# Supplementary material for: Trends in estrogen replacement therapy use among younger gynecologic cancer survivors, 2013-2021
Source: Gynecol Oncol Rep. 2026 Aug 5;66:102178. doi: 10.1016/j.gore.2026.102178 (PMC13476577; doi:10.1016/j.gore.2026.102178)
Supplement: Supplementary file 2 [file mmc2.docx]

| **Brand** | **Generic** | **NDC** | **Strength** | **Dosage Form** | **Route** | **Product NDC** | **11-digit NDC** | **Classification** |
| --- | --- | --- | --- | --- | --- | --- | --- | --- |
| ABIGALE | estradiol and norethindrone acetate | 70700-330-85 | 1mg/1, .5 mg/1 | TABLET | ORAL | 70700-330 | 70700-0330-85 | Combined estrogen–progestogen therapy |
| ABIGALE LO | estradiol and norethindrone acetate | 70700-303-85 | .5mg/1, .1 mg/1 | TABLET | ORAL | 70700-303 | 70700-0303-85 | Combined estrogen–progestogen therapy |
| Activella | Estradiol/norethindrone acetate | 60846-231-01 | .5mg/1, .1 mg/1 | TABLET, FILM COATED | ORAL | 60846-231 | 60846-0231-01 | Combined estrogen–progestogen therapy |
| Activella | Estradiol/norethindrone acetate | 60846-232-01 | 1mg/1, .5 mg/1 | TABLET, FILM COATED | ORAL | 60846-232 | 60846-0232-01 | Combined estrogen–progestogen therapy |
| Angeliq | drospirenone and estradiol | 50419-482-03 | .25mg/1, .5 mg/1 | TABLET, FILM COATED | ORAL | 50419-482 | 50419-0482-03 | Combined estrogen–progestogen therapy |
| Angeliq | drospirenone and estradiol | 50419-482-72 | .25mg/1, .5 mg/1 | TABLET, FILM COATED | ORAL | 50419-482 | 50419-0482-72 | Combined estrogen–progestogen therapy |
| Angeliq | drospirenone and estradiol | 50419-483-03 | .5mg/1, 1 mg/1 | TABLET, FILM COATED | ORAL | 50419-483 | 50419-0483-03 | Combined estrogen–progestogen therapy |
| Angeliq | drospirenone and estradiol | 50419-483-72 | .5mg/1, 1 mg/1 | TABLET, FILM COATED | ORAL | 50419-483 | 50419-0483-72 | Combined estrogen–progestogen therapy |
| Bijuva | estradiol and progesterone | 50261-211-05 | 1mg/1, 100 mg/1 | CAPSULE | ORAL | 50261-211 | 50261-0211-05 | Combined estrogen–progestogen therapy |
| Bijuva | estradiol and progesterone | 50261-251-05 | .5mg/1, 100 mg/1 | CAPSULE | ORAL | 50261-251 | 50261-0251-05 | Combined estrogen–progestogen therapy |
| Bijuva | estradiol and progesterone | 50261-251-30 | .5mg/1, 100 mg/1 | CAPSULE | ORAL | 50261-251 | 50261-0251-30 | Combined estrogen–progestogen therapy |
| Bijuva | estradiol and progesterone | 68308-750-00 | 1mg/1, 100 mg/1 | CAPSULE | ORAL | 68308-750 | 68308-0750-00 | Combined estrogen–progestogen therapy |
| Bijuva | estradiol and progesterone | 68308-750-30 | 1mg/1, 100 mg/1 | CAPSULE | ORAL | 68308-750 | 68308-0750-30 | Combined estrogen–progestogen therapy |
| Bijuva | estradiol and progesterone | 68308-751-00 | .5mg/1, 100 mg/1 | CAPSULE | ORAL | 68308-751 | 68308-0751-00 | Combined estrogen–progestogen therapy |
| Bijuva | estradiol and progesterone | 68308-751-30 | .5mg/1, 100 mg/1 | CAPSULE | ORAL | 68308-751 | 68308-0751-30 | Combined estrogen–progestogen therapy |
| Climara | Estradiol | 50419-451-04 | .05mg/d | PATCH | TRANSDERMAL | 50419-451 | 50419-0451-04 | Transdermal estrogen |
| Climara | Estradiol | 50419-452-04 | .1mg/d | PATCH | TRANSDERMAL | 50419-452 | 50419-0452-04 | Transdermal estrogen |
| Climara | Estradiol | 50419-453-04 | .075mg/d | PATCH | TRANSDERMAL | 50419-453 | 50419-0453-04 | Transdermal estrogen |
| Climara | Estradiol | 50419-454-04 | .025mg/d | PATCH | TRANSDERMAL | 50419-454 | 50419-0454-04 | Transdermal estrogen |
| Climara | Estradiol | 50419-456-04 | .0375mg/d | PATCH | TRANSDERMAL | 50419-456 | 50419-0456-04 | Transdermal estrogen |
| Climara | Estradiol | 50419-459-04 | .06mg/d | PATCH | TRANSDERMAL | 50419-459 | 50419-0459-04 | Transdermal estrogen |
| Climara Pro | Estradiol and Levonorgestrel | 50419-491-04 | .045mg/d, .015 mg/d | PATCH | TRANSDERMAL | 50419-491 | 50419-0491-04 | Transdermal estrogen |
| Climara Pro | Estradiol and Levonorgestrel | 50419-491-73 | .045mg/d, .015 mg/d | PATCH | TRANSDERMAL | 50419-491 | 50419-0491-73 | Transdermal estrogen |
| CombiPatch (estradiol/norethindrone acetate transdermal system) | estradiol/norethindrone acetate transdermal system | 68968-0514-8 | .05mg/d, .14 mg/d | PATCH, EXTENDED RELEASE | TRANSDERMAL | 68968-0514 | 68968-0514-08 | Transdermal estrogen |
| CombiPatch (estradiol/norethindrone acetate transdermal system) | estradiol/norethindrone acetate transdermal system | 68968-0525-8 | .05mg/d, .25 mg/d | PATCH, EXTENDED RELEASE | TRANSDERMAL | 68968-0525 | 68968-0525-08 | Transdermal estrogen |
| Delestrogen | estradiol valerate | 42023-110-01 | 10mg/mL | INJECTION | INTRAMUSCULAR | 42023-110 | 42023-0110-01 | Intramuscular estrogen |
| Delestrogen | estradiol valerate | 42023-111-01 | 20mg/mL | INJECTION | INTRAMUSCULAR | 42023-111 | 42023-0111-01 | Intramuscular estrogen |
| Depo-Estradiol | estradiol cypionate | 0009-0271-01 | 5mg/mL | INJECTION | INTRAMUSCULAR | 0009-0271 | 00009-0271-01 | Intramuscular estrogen |
| DIVIGEL | estradiol | 68025-065-07 | .25mg/.25g | GEL | TOPICAL | 68025-065 | 68025-0065-07 | Transdermal estrogen |
| DIVIGEL | estradiol | 68025-065-30 | .25mg/.25g | GEL | TOPICAL | 68025-065 | 68025-0065-30 | Transdermal estrogen |
| DIVIGEL | estradiol | 68025-066-07 | .5mg/.5g | GEL | TOPICAL | 68025-066 | 68025-0066-07 | Transdermal estrogen |
| DIVIGEL | estradiol | 68025-066-30 | .5mg/.5g | GEL | TOPICAL | 68025-066 | 68025-0066-30 | Transdermal estrogen |
| DIVIGEL | estradiol | 68025-067-07 | 1mg/g | GEL | TOPICAL | 68025-067 | 68025-0067-07 | Transdermal estrogen |
| DIVIGEL | estradiol | 68025-067-30 | 1mg/g | GEL | TOPICAL | 68025-067 | 68025-0067-30 | Transdermal estrogen |
| DIVIGEL | estradiol | 68025-083-07 | .75mg/.75g | GEL | TOPICAL | 68025-083 | 68025-0083-07 | Transdermal estrogen |
| DIVIGEL | estradiol | 68025-083-30 | .75mg/.75g | GEL | TOPICAL | 68025-083 | 68025-0083-30 | Transdermal estrogen |
| DIVIGEL | estradiol | 68025-086-07 | 1.25mg/1.25g | GEL | TOPICAL | 68025-086 | 68025-0086-07 | Transdermal estrogen |
| DIVIGEL | estradiol | 68025-086-30 | 1.25mg/1.25g | GEL | TOPICAL | 68025-086 | 68025-0086-30 | Transdermal estrogen |
| DOTTI | Estradiol | 65162-989-08 | .025mg/d | PATCH, EXTENDED RELEASE | TRANSDERMAL | 65162-989 | 65162-0989-08 | Transdermal estrogen |
| DOTTI | Estradiol | 65162-992-08 | .0375mg/d | PATCH, EXTENDED RELEASE | TRANSDERMAL | 65162-992 | 65162-0992-08 | Transdermal estrogen |
| DOTTI | Estradiol | 65162-993-08 | .05mg/d | PATCH, EXTENDED RELEASE | TRANSDERMAL | 65162-993 | 65162-0993-08 | Transdermal estrogen |
| DOTTI | Estradiol | 65162-995-08 | .075mg/d | PATCH, EXTENDED RELEASE | TRANSDERMAL | 65162-995 | 65162-0995-08 | Transdermal estrogen |
| DOTTI | Estradiol | 65162-997-08 | .1mg/d | PATCH, EXTENDED RELEASE | TRANSDERMAL | 65162-997 | 65162-0997-08 | Transdermal estrogen |
| DOTTI | Estradiol | 72162-2032-2 | .025mg/d | PATCH, EXTENDED RELEASE | TRANSDERMAL | 72162-2032 | 72162-2032-02 | Transdermal estrogen |
| DOTTI | Estradiol | 72162-2033-2 | .0375mg/d | PATCH, EXTENDED RELEASE | TRANSDERMAL | 72162-2033 | 72162-2033-02 | Transdermal estrogen |
| DOTTI | Estradiol | 72162-2034-2 | .05mg/d | PATCH, EXTENDED RELEASE | TRANSDERMAL | 72162-2034 | 72162-2034-02 | Transdermal estrogen |
| DOTTI | Estradiol | 72162-2035-2 | .075mg/d | PATCH, EXTENDED RELEASE | TRANSDERMAL | 72162-2035 | 72162-2035-02 | Transdermal estrogen |
| DOTTI | Estradiol | 72162-2036-2 | .1mg/d | PATCH, EXTENDED RELEASE | TRANSDERMAL | 72162-2036 | 72162-2036-02 | Transdermal estrogen |
| ELESTRIN | ESTRADIOL | 0037-4801-70 | .52mg/.87g | GEL, METERED | TOPICAL | 0037-4801 | 00037-4801-70 | Oral estradiol |
| Estrace | Estradiol | 0430-0720-24 | .5mg/1 | TABLET | ORAL | 0430-0720 | 00430-0720-24 | Oral estradiol |
| Estrace | Estradiol | 0430-0721-24 | 1mg/1 | TABLET | ORAL | 0430-0721 | 00430-0721-24 | Oral estradiol |
| Estrace | Estradiol | 0430-0722-24 | 2mg/1 | TABLET | ORAL | 0430-0722 | 00430-0722-24 | Oral estradiol |
| ESTRACE | estradiol | 0430-3754-14 | .1mg/g | CREAM | VAGINAL | 0430-3754 | 00430-3754-14 | Vaginal estrogen |
| ESTRACE | estradiol | 50090-2321-0 | .1mg/g | CREAM | VAGINAL | 50090-2321 | 50090-2321-00 | Vaginal estrogen |
| Estradiol | Estradiol | 0093-3223-08 | 10ug/1 | INSERT | VAGINAL | 0093-3223 | 00093-3223-08 | Vaginal estrogen |
| Estradiol | Estradiol | 0093-3223-97 | 10ug/1 | INSERT | VAGINAL | 0093-3223 | 00093-3223-97 | Vaginal estrogen |
| Estradiol | Estradiol | 0093-3541-43 | .1mg/g | CREAM | VAGINAL | 0093-3541 | 00093-3541-43 | Vaginal estrogen |
| Estradiol | estradiol | 0378-3349-99 | .025mg/d | PATCH | TRANSDERMAL | 0378-3349 | 00378-3349-99 | Transdermal estrogen |
| Estradiol | estradiol | 0378-3350-99 | .05mg/d | PATCH | TRANSDERMAL | 0378-3350 | 00378-3350-99 | Transdermal estrogen |
| Estradiol | estradiol | 0378-3351-99 | .075mg/d | PATCH | TRANSDERMAL | 0378-3351 | 00378-3351-99 | Transdermal estrogen |
| Estradiol | estradiol | 0378-3352-99 | .1mg/d | PATCH | TRANSDERMAL | 0378-3352 | 00378-3352-99 | Transdermal estrogen |
| Estradiol | estradiol | 0378-3360-99 | .0375mg/d | PATCH | TRANSDERMAL | 0378-3360 | 00378-3360-99 | Transdermal estrogen |
| Estradiol | estradiol | 0378-3361-99 | .06mg/d | PATCH | TRANSDERMAL | 0378-3361 | 00378-3361-99 | Transdermal estrogen |
| Estradiol | estradiol | 0378-4619-26 | .025mg/d | PATCH | TRANSDERMAL | 0378-4619 | 00378-4619-26 | Transdermal estrogen |
| Estradiol | estradiol | 0378-4620-26 | .0375mg/d | PATCH | TRANSDERMAL | 0378-4620 | 00378-4620-26 | Transdermal estrogen |
| Estradiol | estradiol | 0378-4621-26 | .05mg/d | PATCH | TRANSDERMAL | 0378-4621 | 00378-4621-26 | Transdermal estrogen |
| Estradiol | estradiol | 0378-4622-26 | .075mg/d | PATCH | TRANSDERMAL | 0378-4622 | 00378-4622-26 | Transdermal estrogen |
| Estradiol | estradiol | 0378-4623-26 | .1mg/d | PATCH | TRANSDERMAL | 0378-4623 | 00378-4623-26 | Transdermal estrogen |
| Estradiol | estradiol | 0378-4640-26 | .1mg/d | PATCH | TRANSDERMAL | 0378-4640 | 00378-4640-26 | Transdermal estrogen |
| Estradiol | estradiol | 0378-4641-26 | .075mg/d | PATCH | TRANSDERMAL | 0378-4641 | 00378-4641-26 | Transdermal estrogen |
| Estradiol | estradiol | 0378-4642-26 | .05mg/d | PATCH | TRANSDERMAL | 0378-4642 | 00378-4642-26 | Transdermal estrogen |
| Estradiol | estradiol | 0378-4643-26 | .0375mg/d | PATCH | TRANSDERMAL | 0378-4643 | 00378-4643-26 | Transdermal estrogen |
| Estradiol | estradiol | 0378-4644-26 | .025mg/d | PATCH | TRANSDERMAL | 0378-4644 | 00378-4644-26 | Transdermal estrogen |
| Estradiol | Estradiol | 0378-8770-35 | .1mg/g | CREAM | VAGINAL | 0378-8770 | 00378-8770-35 | Vaginal estrogen |
| Estradiol | Estradiol | 0555-0886-02 | 1mg/1 | TABLET | ORAL | 0555-0886 | 00555-0886-02 | Oral estradiol |
| Estradiol | Estradiol | 0555-0886-04 | 1mg/1 | TABLET | ORAL | 0555-0886 | 00555-0886-04 | Oral estradiol |
| Estradiol | Estradiol | 0555-0887-02 | 2mg/1 | TABLET | ORAL | 0555-0887 | 00555-0887-02 | Oral estradiol |
| Estradiol | Estradiol | 0555-0887-04 | 2mg/1 | TABLET | ORAL | 0555-0887 | 00555-0887-04 | Oral estradiol |
| Estradiol | Estradiol | 0555-0899-02 | .5mg/1 | TABLET | ORAL | 0555-0899 | 00555-0899-02 | Oral estradiol |
| Estradiol | estradiol | 0781-7129-83 | .025mg/d | PATCH, EXTENDED RELEASE | TRANSDERMAL | 0781-7129 | 00781-7129-83 | Transdermal estrogen |
| Estradiol | estradiol | 0781-7138-83 | .0375mg/d | PATCH, EXTENDED RELEASE | TRANSDERMAL | 0781-7138 | 00781-7138-83 | Transdermal estrogen |
| Estradiol | estradiol | 0781-7144-83 | .05mg/d | PATCH, EXTENDED RELEASE | TRANSDERMAL | 0781-7144 | 00781-7144-83 | Transdermal estrogen |
| Estradiol | estradiol | 0781-7156-83 | .075mg/d | PATCH, EXTENDED RELEASE | TRANSDERMAL | 0781-7156 | 00781-7156-83 | Transdermal estrogen |
| Estradiol | estradiol | 0781-7167-83 | .1mg/d | PATCH, EXTENDED RELEASE | TRANSDERMAL | 0781-7167 | 00781-7167-83 | Transdermal estrogen |
| Estradiol | Estradiol | 13811-090-32 | .25mg/.25g | GEL | TOPICAL | 13811-090 | 13811-0090-32 | Transdermal estrogen |
| Estradiol | Estradiol | 13811-091-32 | .5mg/.5g | GEL | TOPICAL | 13811-091 | 13811-0091-32 | Transdermal estrogen |
| Estradiol | Estradiol | 13811-092-32 | .75mg/.75g | GEL | TOPICAL | 13811-092 | 13811-0092-32 | Transdermal estrogen |
| Estradiol | Estradiol | 13811-093-32 | 1mg/g | GEL | TOPICAL | 13811-093 | 13811-0093-32 | Transdermal estrogen |
| Estradiol | Estradiol | 13811-094-32 | 1.25mg/1.25g | GEL | TOPICAL | 13811-094 | 13811-0094-32 | Transdermal estrogen |
| ESTRADIOL | ESTRADIOL | 24658-702-01 | .5mg/1 | TABLET | ORAL | 24658-702 | 24658-0702-01 | Oral estradiol |
| ESTRADIOL | ESTRADIOL | 24658-702-05 | .5mg/1 | TABLET | ORAL | 24658-702 | 24658-0702-05 | Oral estradiol |
| ESTRADIOL | ESTRADIOL | 24658-703-01 | 1mg/1 | TABLET | ORAL | 24658-703 | 24658-0703-01 | Oral estradiol |
| ESTRADIOL | ESTRADIOL | 24658-703-05 | 1mg/1 | TABLET | ORAL | 24658-703 | 24658-0703-05 | Oral estradiol |
| ESTRADIOL | ESTRADIOL | 24658-704-01 | 2mg/1 | TABLET | ORAL | 24658-704 | 24658-0704-01 | Oral estradiol |
| ESTRADIOL | ESTRADIOL | 24658-704-05 | 2mg/1 | TABLET | ORAL | 24658-704 | 24658-0704-05 | Oral estradiol |
| ESTRADIOL | ESTRADIOL | 24658-704-90 | 2mg/1 | TABLET | ORAL | 24658-704 | 24658-0704-90 | Oral estradiol |
| ESTRADIOL | ESTRADIOL | 42806-087-01 | .5mg/1 | TABLET | ORAL | 42806-087 | 42806-0087-01 | Oral estradiol |
| ESTRADIOL | ESTRADIOL | 42806-087-05 | .5mg/1 | TABLET | ORAL | 42806-087 | 42806-0087-05 | Oral estradiol |
| ESTRADIOL | ESTRADIOL | 42806-088-01 | 1mg/1 | TABLET | ORAL | 42806-088 | 42806-0088-01 | Oral estradiol |
| ESTRADIOL | ESTRADIOL | 42806-088-05 | 1mg/1 | TABLET | ORAL | 42806-088 | 42806-0088-05 | Oral estradiol |
| ESTRADIOL | ESTRADIOL | 42806-089-01 | 2mg/1 | TABLET | ORAL | 42806-089 | 42806-0089-01 | Oral estradiol |
| ESTRADIOL | ESTRADIOL | 42806-089-05 | 2mg/1 | TABLET | ORAL | 42806-089 | 42806-0089-05 | Oral estradiol |
| ESTRADIOL | ESTRADIOL | 42806-089-09 | 2mg/1 | TABLET | ORAL | 42806-089 | 42806-0089-09 | Oral estradiol |
| ESTRADIOL | estradiol | 45802-134-30 | .25mg/.25g | GEL | TOPICAL | 45802-134 | 45802-0134-30 | Transdermal estrogen |
| ESTRADIOL | estradiol | 45802-202-30 | .5mg/.5g | GEL | TOPICAL | 45802-202 | 45802-0202-30 | Transdermal estrogen |
| ESTRADIOL | estradiol | 45802-310-30 | .75mg/.75g | GEL | TOPICAL | 45802-310 | 45802-0310-30 | Transdermal estrogen |
| ESTRADIOL | estradiol | 45802-452-30 | 1mg/g | GEL | TOPICAL | 45802-452 | 45802-0452-30 | Transdermal estrogen |
| ESTRADIOL | estradiol | 45802-573-30 | 1.25mg/1.25g | GEL | TOPICAL | 45802-573 | 45802-0573-30 | Transdermal estrogen |
| Estradiol | Estradiol | 47781-104-44 | .1mg/g | CREAM | VAGINAL | 47781-104 | 47781-0104-44 | Vaginal estrogen |
| Estradiol | Estradiol | 50090-1704-0 | 1mg/1 | TABLET | ORAL | 50090-1704 | 50090-1704-00 | Oral estradiol |
| Estradiol | Estradiol | 50090-1704-1 | 1mg/1 | TABLET | ORAL | 50090-1704 | 50090-1704-01 | Oral estradiol |
| Estradiol | Estradiol | 50090-1704-2 | 1mg/1 | TABLET | ORAL | 50090-1704 | 50090-1704-02 | Oral estradiol |
| Estradiol | Estradiol | 50090-1876-0 | 2mg/1 | TABLET | ORAL | 50090-1876 | 50090-1876-00 | Oral estradiol |
| Estradiol | Estradiol | 50090-1876-2 | 2mg/1 | TABLET | ORAL | 50090-1876 | 50090-1876-02 | Oral estradiol |
| Estradiol | Estradiol | 50090-1882-0 | .5mg/1 | TABLET | ORAL | 50090-1882 | 50090-1882-00 | Oral estradiol |
| Estradiol | Estradiol | 50090-1882-1 | .5mg/1 | TABLET | ORAL | 50090-1882 | 50090-1882-01 | Oral estradiol |
| Estradiol | Estradiol | 50090-3327-0 | .1mg/g | CREAM | VAGINAL | 50090-3327 | 50090-3327-00 | Vaginal estrogen |
| Estradiol | Estradiol | 50090-3645-0 | .1mg/g | CREAM | VAGINAL | 50090-3645 | 50090-3645-00 | Vaginal estrogen |
| ESTRADIOL | ESTRADIOL | 50090-5494-0 | .5mg/1 | TABLET | ORAL | 50090-5494 | 50090-5494-00 | Oral estradiol |
| ESTRADIOL | ESTRADIOL | 50090-5494-1 | .5mg/1 | TABLET | ORAL | 50090-5494 | 50090-5494-01 | Oral estradiol |
| ESTRADIOL | ESTRADIOL | 50090-6359-0 | 1mg/1 | TABLET | ORAL | 50090-6359 | 50090-6359-00 | Oral estradiol |
| ESTRADIOL | ESTRADIOL | 50090-6359-2 | 1mg/1 | TABLET | ORAL | 50090-6359 | 50090-6359-02 | Oral estradiol |
| ESTRADIOL | ESTRADIOL | 50090-6363-0 | 2mg/1 | TABLET | ORAL | 50090-6363 | 50090-6363-00 | Oral estradiol |
| ESTRADIOL | ESTRADIOL | 50090-6363-2 | 2mg/1 | TABLET | ORAL | 50090-6363 | 50090-6363-02 | Oral estradiol |
| Estradiol | Estradiol | 50090-6847-0 | 1mg/1 | TABLET | ORAL | 50090-6847 | 50090-6847-00 | Oral estradiol |
| ESTRADIOL | ESTRADIOL | 50090-7452-0 | 1mg/1 | TABLET | ORAL | 50090-7452 | 50090-7452-00 | Oral estradiol |
| Estradiol | Estradiol | 50090-7480-0 | 1mg/1 | TABLET | ORAL | 50090-7480 | 50090-7480-00 | Oral estradiol |
| Estradiol | Estradiol | 50090-7480-2 | 1mg/1 | TABLET | ORAL | 50090-7480 | 50090-7480-02 | Oral estradiol |
| Estradiol | Estradiol | 50090-7481-0 | 1mg/1 | TABLET | ORAL | 50090-7481 | 50090-7481-00 | Oral estradiol |
| Estradiol | Estradiol | 51655-227-26 | 2mg/1 | TABLET | ORAL | 51655-227 | 51655-0227-26 | Oral estradiol |
| Estradiol | Estradiol | 51655-227-52 | 2mg/1 | TABLET | ORAL | 51655-227 | 51655-0227-52 | Oral estradiol |
| Estradiol | Estradiol | 51655-229-26 | 1mg/1 | TABLET | ORAL | 51655-229 | 51655-0229-26 | Oral estradiol |
| Estradiol | Estradiol | 51655-229-52 | 1mg/1 | TABLET | ORAL | 51655-229 | 51655-0229-52 | Oral estradiol |
| ESTRADIOL | ESTRADIOL | 51655-860-26 | .5mg/1 | TABLET | ORAL | 51655-860 | 51655-0860-26 | Oral estradiol |
| ESTRADIOL | ESTRADIOL | 51655-860-52 | .5mg/1 | TABLET | ORAL | 51655-860 | 51655-0860-52 | Oral estradiol |
| Estradiol | Estradiol | 53746-226-21 | 10ug/1 | INSERT | VAGINAL | 53746-226 | 53746-0226-21 | Vaginal estrogen |
| Estradiol | Estradiol | 53746-226-23 | 10ug/1 | INSERT | VAGINAL | 53746-226 | 53746-0226-23 | Vaginal estrogen |
| Estradiol | ESTRADIOL | 54348-701-30 | 1mg/1 | TABLET | ORAL | 54348-701 | 54348-0701-30 | Oral estradiol |
| Estradiol | ESTRADIOL | 54348-702-90 | 2mg/1 | TABLET | ORAL | 54348-702 | 54348-0702-90 | Oral estradiol |
| Estradiol | Estradiol | 60429-834-05 | 1mg/1 | TABLET | ORAL | 60429-834 | 60429-0834-05 | Oral estradiol |
| Estradiol | Estradiol | 63187-741-30 | 1mg/1 | TABLET | ORAL | 63187-741 | 63187-0741-30 | Oral estradiol |
| Estradiol | Estradiol | 63187-741-60 | 1mg/1 | TABLET | ORAL | 63187-741 | 63187-0741-60 | Oral estradiol |
| Estradiol | Estradiol | 63187-741-90 | 1mg/1 | TABLET | ORAL | 63187-741 | 63187-0741-90 | Oral estradiol |
| ESTRADIOL | ESTRADIOL | 63629-4788-1 | 2mg/1 | TABLET | ORAL | 63629-4788 | 63629-4788-01 | Oral estradiol |
| ESTRADIOL | ESTRADIOL | 63629-4788-2 | 2mg/1 | TABLET | ORAL | 63629-4788 | 63629-4788-02 | Oral estradiol |
| ESTRADIOL | ESTRADIOL | 63629-4788-3 | 2mg/1 | TABLET | ORAL | 63629-4788 | 63629-4788-03 | Oral estradiol |
| ESTRADIOL | ESTRADIOL | 63629-4788-4 | 2mg/1 | TABLET | ORAL | 63629-4788 | 63629-4788-04 | Oral estradiol |
| ESTRADIOL | ESTRADIOL | 63629-4788-5 | 2mg/1 | TABLET | ORAL | 63629-4788 | 63629-4788-05 | Oral estradiol |
| Estradiol | Estradiol | 63629-8713-1 | .1mg/g | CREAM | VAGINAL | 63629-8713 | 63629-8713-01 | Vaginal estrogen |
| Estradiol | Estradiol | 63629-8802-1 | .5mg/1 | TABLET | ORAL | 63629-8802 | 63629-8802-01 | Oral estradiol |
| Estradiol | Estradiol | 63629-8803-1 | 10ug/1 | INSERT | VAGINAL | 63629-8803 | 63629-8803-01 | Vaginal estrogen |
| Estradiol | Estradiol | 63629-8804-1 | 10ug/1 | INSERT | VAGINAL | 63629-8804 | 63629-8804-01 | Vaginal estrogen |
| Estradiol | Estradiol | 68071-2675-3 | 2mg/1 | TABLET | ORAL | 68071-2675 | 68071-2675-03 | Oral estradiol |
| Estradiol | Estradiol | 68071-2675-9 | 2mg/1 | TABLET | ORAL | 68071-2675 | 68071-2675-09 | Oral estradiol |
| ESTRADIOL | ESTRADIOL | 68071-3394-2 | 1mg/1 | TABLET | ORAL | 68071-3394 | 68071-3394-02 | Oral estradiol |
| ESTRADIOL | ESTRADIOL | 68071-3394-3 | 1mg/1 | TABLET | ORAL | 68071-3394 | 68071-3394-03 | Oral estradiol |
| ESTRADIOL | ESTRADIOL | 68071-3394-6 | 1mg/1 | TABLET | ORAL | 68071-3394 | 68071-3394-06 | Oral estradiol |
| ESTRADIOL | ESTRADIOL | 68071-3394-9 | 1mg/1 | TABLET | ORAL | 68071-3394 | 68071-3394-09 | Oral estradiol |
| Estradiol | Estradiol | 68071-3629-3 | 1mg/1 | TABLET | ORAL | 68071-3629 | 68071-3629-03 | Oral estradiol |
| Estradiol | Estradiol | 68071-3689-9 | 2mg/1 | TABLET | ORAL | 68071-3689 | 68071-3689-09 | Oral estradiol |
| ESTRADIOL | ESTRADIOL | 68071-4485-1 | 2mg/1 | TABLET | ORAL | 68071-4485 | 68071-4485-01 | Oral estradiol |
| ESTRADIOL | ESTRADIOL | 68071-4485-3 | 2mg/1 | TABLET | ORAL | 68071-4485 | 68071-4485-03 | Oral estradiol |
| ESTRADIOL | ESTRADIOL | 68071-4485-6 | 2mg/1 | TABLET | ORAL | 68071-4485 | 68071-4485-06 | Oral estradiol |
| ESTRADIOL | ESTRADIOL | 68071-4485-9 | 2mg/1 | TABLET | ORAL | 68071-4485 | 68071-4485-09 | Oral estradiol |
| estradiol | estradiol | 68382-323-04 | .014mg/d | PATCH | TRANSDERMAL | 68382-323 | 68382-0323-04 | Transdermal estrogen |
| estradiol | estradiol | 68382-324-04 | .025mg/d | PATCH | TRANSDERMAL | 68382-324 | 68382-0324-04 | Transdermal estrogen |
| estradiol | estradiol | 68382-325-04 | .0375mg/d | PATCH | TRANSDERMAL | 68382-325 | 68382-0325-04 | Transdermal estrogen |
| estradiol | estradiol | 68382-326-04 | .05mg/d | PATCH | TRANSDERMAL | 68382-326 | 68382-0326-04 | Transdermal estrogen |
| estradiol | estradiol | 68382-327-04 | .06mg/d | PATCH | TRANSDERMAL | 68382-327 | 68382-0327-04 | Transdermal estrogen |
| estradiol | estradiol | 68382-328-04 | .075mg/d | PATCH | TRANSDERMAL | 68382-328 | 68382-0328-04 | Transdermal estrogen |
| estradiol | estradiol | 68382-329-04 | .1mg/d | PATCH | TRANSDERMAL | 68382-329 | 68382-0329-04 | Transdermal estrogen |
| ESTRADIOL | ESTRADIOL | 68788-7102-1 | 1mg/1 | TABLET | ORAL | 68788-7102 | 68788-7102-01 | Oral estradiol |
| ESTRADIOL | ESTRADIOL | 68788-7102-3 | 1mg/1 | TABLET | ORAL | 68788-7102 | 68788-7102-03 | Oral estradiol |
| ESTRADIOL | ESTRADIOL | 68788-7102-6 | 1mg/1 | TABLET | ORAL | 68788-7102 | 68788-7102-06 | Oral estradiol |
| ESTRADIOL | ESTRADIOL | 68788-7102-9 | 1mg/1 | TABLET | ORAL | 68788-7102 | 68788-7102-09 | Oral estradiol |
| ESTRADIOL | ESTRADIOL | 68788-7332-1 | .5mg/1 | TABLET | ORAL | 68788-7332 | 68788-7332-01 | Oral estradiol |
| ESTRADIOL | ESTRADIOL | 68788-7332-3 | .5mg/1 | TABLET | ORAL | 68788-7332 | 68788-7332-03 | Oral estradiol |
| ESTRADIOL | ESTRADIOL | 68788-7332-6 | .5mg/1 | TABLET | ORAL | 68788-7332 | 68788-7332-06 | Oral estradiol |
| ESTRADIOL | ESTRADIOL | 68788-7332-9 | .5mg/1 | TABLET | ORAL | 68788-7332 | 68788-7332-09 | Oral estradiol |
| Estradiol | Estradiol | 68788-8481-1 | .5mg/1 | TABLET | ORAL | 68788-8481 | 68788-8481-01 | Oral estradiol |
| Estradiol | Estradiol | 68788-8481-3 | .5mg/1 | TABLET | ORAL | 68788-8481 | 68788-8481-03 | Oral estradiol |
| Estradiol | Estradiol | 68788-8481-6 | .5mg/1 | TABLET | ORAL | 68788-8481 | 68788-8481-06 | Oral estradiol |
| Estradiol | Estradiol | 68788-8481-9 | .5mg/1 | TABLET | ORAL | 68788-8481 | 68788-8481-09 | Oral estradiol |
| Estradiol | Estradiol | 68788-8566-8 | .0375mg/d | PATCH, EXTENDED RELEASE | TRANSDERMAL | 68788-8566 | 68788-8566-08 | Transdermal estrogen |
| Estradiol | Estradiol | 68788-8570-8 | .05mg/d | PATCH, EXTENDED RELEASE | TRANSDERMAL | 68788-8570 | 68788-8570-08 | Transdermal estrogen |
| Estradiol | Estradiol | 68788-8626-1 | .5mg/1 | TABLET | ORAL | 68788-8626 | 68788-8626-01 | Oral estradiol |
| Estradiol | Estradiol | 68788-8626-3 | .5mg/1 | TABLET | ORAL | 68788-8626 | 68788-8626-03 | Oral estradiol |
| Estradiol | Estradiol | 68788-8626-6 | .5mg/1 | TABLET | ORAL | 68788-8626 | 68788-8626-06 | Oral estradiol |
| Estradiol | Estradiol | 68788-8626-9 | .5mg/1 | TABLET | ORAL | 68788-8626 | 68788-8626-09 | Oral estradiol |
| Estradiol | estradiol | 68968-3410-8 | .1mg/d | FILM, EXTENDED RELEASE | TRANSDERMAL | 68968-3410 | 68968-3410-08 | Transdermal estrogen |
| Estradiol | estradiol | 68968-3425-8 | .025mg/d | FILM, EXTENDED RELEASE | TRANSDERMAL | 68968-3425 | 68968-3425-08 | Transdermal estrogen |
| Estradiol | estradiol | 68968-3437-8 | .0375mg/d | FILM, EXTENDED RELEASE | TRANSDERMAL | 68968-3437 | 68968-3437-08 | Transdermal estrogen |
| Estradiol | estradiol | 68968-3450-8 | .05mg/d | FILM, EXTENDED RELEASE | TRANSDERMAL | 68968-3450 | 68968-3450-08 | Transdermal estrogen |
| Estradiol | estradiol | 68968-3475-8 | .075mg/d | FILM, EXTENDED RELEASE | TRANSDERMAL | 68968-3475 | 68968-3475-08 | Transdermal estrogen |
| Estradiol | Estradiol | 69238-1293-7 | .0375mg/d | PATCH, EXTENDED RELEASE | TRANSDERMAL | 69238-1293 | 69238-1293-07 | Transdermal estrogen |
| Estradiol | Estradiol | 69238-1294-7 | .075mg/d | PATCH, EXTENDED RELEASE | TRANSDERMAL | 69238-1294 | 69238-1294-07 | Transdermal estrogen |
| Estradiol | Estradiol | 69238-1295-7 | .05mg/d | PATCH, EXTENDED RELEASE | TRANSDERMAL | 69238-1295 | 69238-1295-07 | Transdermal estrogen |
| Estradiol | Estradiol | 69238-1296-7 | .1mg/d | PATCH, EXTENDED RELEASE | TRANSDERMAL | 69238-1296 | 69238-1296-07 | Transdermal estrogen |
| Estradiol | Estradiol | 69238-1447-7 | .025mg/d | PATCH, EXTENDED RELEASE | TRANSDERMAL | 69238-1447 | 69238-1447-07 | Transdermal estrogen |
| Estradiol | Estradiol | 69238-1629-7 | .025mg/d | PATCH, EXTENDED RELEASE | TRANSDERMAL | 69238-1629 | 69238-1629-07 | Transdermal estrogen |
| Estradiol | Estradiol | 69238-1630-7 | .0375mg/d | PATCH, EXTENDED RELEASE | TRANSDERMAL | 69238-1630 | 69238-1630-07 | Transdermal estrogen |
| Estradiol | Estradiol | 69238-1631-7 | .05mg/d | PATCH, EXTENDED RELEASE | TRANSDERMAL | 69238-1631 | 69238-1631-07 | Transdermal estrogen |
| Estradiol | Estradiol | 69238-1632-7 | .075mg/d | PATCH, EXTENDED RELEASE | TRANSDERMAL | 69238-1632 | 69238-1632-07 | Transdermal estrogen |
| Estradiol | Estradiol | 69238-1633-7 | .1mg/d | PATCH, EXTENDED RELEASE | TRANSDERMAL | 69238-1633 | 69238-1633-07 | Transdermal estrogen |
| Estradiol | Estradiol Transdermal | 69238-2448-3 | 1.25mg/1.25g | GEL | TOPICAL | 69238-2448 | 69238-2448-03 | Transdermal estrogen |
| Estradiol | Estradiol Transdermal | 69238-2449-3 | 1mg/g | GEL | TOPICAL | 69238-2449 | 69238-2449-03 | Transdermal estrogen |
| Estradiol | Estradiol Transdermal | 69238-2450-3 | .75mg/.75g | GEL | TOPICAL | 69238-2450 | 69238-2450-03 | Transdermal estrogen |
| Estradiol | Estradiol Transdermal | 69238-2451-3 | .5mg/.5g | GEL | TOPICAL | 69238-2451 | 69238-2451-03 | Transdermal estrogen |
| Estradiol | Estradiol Transdermal | 69238-2452-3 | .25mg/.25g | GEL | TOPICAL | 69238-2452 | 69238-2452-03 | Transdermal estrogen |
| Estradiol | Estradiol | 70518-0834-0 | 1mg/1 | TABLET | ORAL | 70518-0834 | 70518-0834-00 | Oral estradiol |
| Estradiol | Estradiol | 70518-0920-0 | 2mg/1 | TABLET | ORAL | 70518-0920 | 70518-0920-00 | Oral estradiol |
| ESTRADIOL | ESTRADIOL | 70518-2829-0 | 2mg/1 | TABLET | ORAL | 70518-2829 | 70518-2829-00 | Oral estradiol |
| ESTRADIOL | ESTRADIOL | 70518-3818-0 | 1mg/1 | TABLET | ORAL | 70518-3818 | 70518-3818-00 | Oral estradiol |
| Estradiol | Estradiol | 70518-3910-0 | 2mg/1 | TABLET | ORAL | 70518-3910 | 70518-3910-00 | Oral estradiol |
| Estradiol | Estradiol | 70518-3910-2 | 2mg/1 | TABLET | ORAL | 70518-3910 | 70518-3910-02 | Oral estradiol |
| Estradiol | Estradiol | 70518-3922-0 | 1mg/1 | TABLET | ORAL | 70518-3922 | 70518-3922-00 | Oral estradiol |
| Estradiol | Estradiol | 70518-3922-1 | 1mg/1 | TABLET | ORAL | 70518-3922 | 70518-3922-01 | Oral estradiol |
| Estradiol | Estradiol Gel 0.1% | 70700-143-35 | .25mg/.25g | GEL | TOPICAL | 70700-143 | 70700-0143-35 | Transdermal estrogen |
| Estradiol | Estradiol Gel 0.1% | 70700-144-35 | .5mg/.5g | GEL | TOPICAL | 70700-144 | 70700-0144-35 | Transdermal estrogen |
| Estradiol | Estradiol Gel 0.1% | 70700-145-35 | 1mg/g | GEL | TOPICAL | 70700-145 | 70700-0145-35 | Transdermal estrogen |
| Estradiol | Estradiol Gel 0.1% | 70700-194-35 | .75mg/.75g | GEL | TOPICAL | 70700-194 | 70700-0194-35 | Transdermal estrogen |
| Estradiol | Estradiol Gel 0.1% | 70700-195-35 | 1.25mg/1.25g | GEL | TOPICAL | 70700-195 | 70700-0195-35 | Transdermal estrogen |
| Estradiol | Estradiol | 70710-1191-8 | .025mg/d | PATCH, EXTENDED RELEASE | TRANSDERMAL | 70710-1191 | 70710-1191-08 | Transdermal estrogen |
| Estradiol | Estradiol | 70710-1192-8 | .0375mg/d | PATCH, EXTENDED RELEASE | TRANSDERMAL | 70710-1192 | 70710-1192-08 | Transdermal estrogen |
| Estradiol | Estradiol | 70710-1193-8 | .05mg/d | PATCH, EXTENDED RELEASE | TRANSDERMAL | 70710-1193 | 70710-1193-08 | Transdermal estrogen |
| Estradiol | Estradiol | 70710-1194-8 | .075mg/d | PATCH, EXTENDED RELEASE | TRANSDERMAL | 70710-1194 | 70710-1194-08 | Transdermal estrogen |
| Estradiol | Estradiol | 70710-1195-8 | .1mg/d | PATCH, EXTENDED RELEASE | TRANSDERMAL | 70710-1195 | 70710-1195-08 | Transdermal estrogen |
| estradiol | estradiol | 70771-1400-4 | .014mg/d | PATCH | TRANSDERMAL | 70771-1400 | 70771-1400-04 | Transdermal estrogen |
| estradiol | estradiol | 70771-1401-4 | .025mg/d | PATCH | TRANSDERMAL | 70771-1401 | 70771-1401-04 | Transdermal estrogen |
| estradiol | estradiol | 70771-1402-4 | .0375mg/d | PATCH | TRANSDERMAL | 70771-1402 | 70771-1402-04 | Transdermal estrogen |
| estradiol | estradiol | 70771-1403-4 | .05mg/d | PATCH | TRANSDERMAL | 70771-1403 | 70771-1403-04 | Transdermal estrogen |
| estradiol | estradiol | 70771-1404-4 | .06mg/d | PATCH | TRANSDERMAL | 70771-1404 | 70771-1404-04 | Transdermal estrogen |
| estradiol | estradiol | 70771-1405-4 | .075mg/d | PATCH | TRANSDERMAL | 70771-1405 | 70771-1405-04 | Transdermal estrogen |
| estradiol | estradiol | 70771-1406-4 | .1mg/d | PATCH | TRANSDERMAL | 70771-1406 | 70771-1406-04 | Transdermal estrogen |
| Estradiol | Estradiol | 70771-1563-8 | .025mg/d | PATCH, EXTENDED RELEASE | TRANSDERMAL | 70771-1563 | 70771-1563-08 | Transdermal estrogen |
| Estradiol | Estradiol | 70771-1564-8 | .0375mg/d | PATCH, EXTENDED RELEASE | TRANSDERMAL | 70771-1564 | 70771-1564-08 | Transdermal estrogen |
| Estradiol | Estradiol | 70771-1565-8 | .05mg/d | PATCH, EXTENDED RELEASE | TRANSDERMAL | 70771-1565 | 70771-1565-08 | Transdermal estrogen |
| Estradiol | Estradiol | 70771-1566-8 | .075mg/d | PATCH, EXTENDED RELEASE | TRANSDERMAL | 70771-1566 | 70771-1566-08 | Transdermal estrogen |
| Estradiol | Estradiol | 70771-1567-8 | .1mg/d | PATCH, EXTENDED RELEASE | TRANSDERMAL | 70771-1567 | 70771-1567-08 | Transdermal estrogen |
| Estradiol | Estradiol | 70954-530-20 | .5mg/.5g | GEL | TOPICAL | 70954-530 | 70954-0530-20 | Transdermal estrogen |
| Estradiol | Estradiol | 70954-531-20 | .25mg/.25g | GEL | TOPICAL | 70954-531 | 70954-0531-20 | Transdermal estrogen |
| Estradiol | Estradiol | 70954-532-20 | .75mg/.75g | GEL | TOPICAL | 70954-532 | 70954-0532-20 | Transdermal estrogen |
| Estradiol | Estradiol | 70954-533-20 | 1mg/g | GEL | TOPICAL | 70954-533 | 70954-0533-20 | Transdermal estrogen |
| Estradiol | Estradiol | 70954-534-20 | 1.25mg/1.25g | GEL | TOPICAL | 70954-534 | 70954-0534-20 | Transdermal estrogen |
| Estradiol | Estradiol | 70954-564-10 | .5mg/1 | TABLET | ORAL | 70954-564 | 70954-0564-10 | Oral estradiol |
| Estradiol | Estradiol | 70954-565-10 | 1mg/1 | TABLET | ORAL | 70954-565 | 70954-0565-10 | Oral estradiol |
| Estradiol | Estradiol | 70954-565-20 | 1mg/1 | TABLET | ORAL | 70954-565 | 70954-0565-20 | Oral estradiol |
| Estradiol | Estradiol | 70954-566-10 | 2mg/1 | TABLET | ORAL | 70954-566 | 70954-0566-10 | Oral estradiol |
| Estradiol | Estradiol | 70954-566-20 | 2mg/1 | TABLET | ORAL | 70954-566 | 70954-0566-20 | Oral estradiol |
| Estradiol | Estradiol | 70954-646-10 | .75mg/1.25g | GEL, METERED | TOPICAL | 70954-646 | 70954-0646-10 | Transdermal estrogen |
| Estradiol | Estradiol | 71335-0201-1 | 2mg/1 | TABLET | ORAL | 71335-0201 | 71335-0201-01 | Oral estradiol |
| Estradiol | Estradiol | 71335-0201-2 | 2mg/1 | TABLET | ORAL | 71335-0201 | 71335-0201-02 | Oral estradiol |
| Estradiol | Estradiol | 71335-0201-3 | 2mg/1 | TABLET | ORAL | 71335-0201 | 71335-0201-03 | Oral estradiol |
| Estradiol | Estradiol | 71335-0201-4 | 2mg/1 | TABLET | ORAL | 71335-0201 | 71335-0201-04 | Oral estradiol |
| Estradiol | Estradiol | 71335-0201-5 | 2mg/1 | TABLET | ORAL | 71335-0201 | 71335-0201-05 | Oral estradiol |
| ESTRADIOL | ESTRADIOL | 71335-0226-1 | .5mg/1 | TABLET | ORAL | 71335-0226 | 71335-0226-01 | Oral estradiol |
| ESTRADIOL | ESTRADIOL | 71335-0226-2 | .5mg/1 | TABLET | ORAL | 71335-0226 | 71335-0226-02 | Oral estradiol |
| ESTRADIOL | ESTRADIOL | 71335-0226-3 | .5mg/1 | TABLET | ORAL | 71335-0226 | 71335-0226-03 | Oral estradiol |
| ESTRADIOL | ESTRADIOL | 71335-0226-4 | .5mg/1 | TABLET | ORAL | 71335-0226 | 71335-0226-04 | Oral estradiol |
| Estradiol | Estradiol | 71335-0430-1 | .5mg/1 | TABLET | ORAL | 71335-0430 | 71335-0430-01 | Oral estradiol |
| Estradiol | Estradiol | 71335-0430-2 | .5mg/1 | TABLET | ORAL | 71335-0430 | 71335-0430-02 | Oral estradiol |
| Estradiol | Estradiol | 71335-0430-3 | .5mg/1 | TABLET | ORAL | 71335-0430 | 71335-0430-03 | Oral estradiol |
| Estradiol | Estradiol | 71335-0430-4 | .5mg/1 | TABLET | ORAL | 71335-0430 | 71335-0430-04 | Oral estradiol |
| Estradiol | Estradiol | 71335-0445-1 | 1mg/1 | TABLET | ORAL | 71335-0445 | 71335-0445-01 | Oral estradiol |
| Estradiol | Estradiol | 71335-0445-2 | 1mg/1 | TABLET | ORAL | 71335-0445 | 71335-0445-02 | Oral estradiol |
| Estradiol | Estradiol | 71335-0445-3 | 1mg/1 | TABLET | ORAL | 71335-0445 | 71335-0445-03 | Oral estradiol |
| Estradiol | Estradiol | 71335-0445-4 | 1mg/1 | TABLET | ORAL | 71335-0445 | 71335-0445-04 | Oral estradiol |
| Estradiol | Estradiol | 71335-0445-5 | 1mg/1 | TABLET | ORAL | 71335-0445 | 71335-0445-05 | Oral estradiol |
| Estradiol | Estradiol | 71335-0445-6 | 1mg/1 | TABLET | ORAL | 71335-0445 | 71335-0445-06 | Oral estradiol |
| Estradiol | Estradiol | 71335-0445-7 | 1mg/1 | TABLET | ORAL | 71335-0445 | 71335-0445-07 | Oral estradiol |
| ESTRADIOL | ESTRADIOL | 71335-0797-1 | 1mg/1 | TABLET | ORAL | 71335-0797 | 71335-0797-01 | Oral estradiol |
| ESTRADIOL | ESTRADIOL | 71335-0797-2 | 1mg/1 | TABLET | ORAL | 71335-0797 | 71335-0797-02 | Oral estradiol |
| ESTRADIOL | ESTRADIOL | 71335-0797-3 | 1mg/1 | TABLET | ORAL | 71335-0797 | 71335-0797-03 | Oral estradiol |
| ESTRADIOL | ESTRADIOL | 71335-0797-4 | 1mg/1 | TABLET | ORAL | 71335-0797 | 71335-0797-04 | Oral estradiol |
| ESTRADIOL | ESTRADIOL | 71335-0797-5 | 1mg/1 | TABLET | ORAL | 71335-0797 | 71335-0797-05 | Oral estradiol |
| ESTRADIOL | ESTRADIOL | 71335-0797-6 | 1mg/1 | TABLET | ORAL | 71335-0797 | 71335-0797-06 | Oral estradiol |
| ESTRADIOL | ESTRADIOL | 71335-0797-7 | 1mg/1 | TABLET | ORAL | 71335-0797 | 71335-0797-07 | Oral estradiol |
| Estradiol | Estradiol | 71335-1719-1 | 2mg/1 | TABLET | ORAL | 71335-1719 | 71335-1719-01 | Oral estradiol |
| Estradiol | Estradiol | 71335-1719-2 | 2mg/1 | TABLET | ORAL | 71335-1719 | 71335-1719-02 | Oral estradiol |
| Estradiol | Estradiol | 71335-1719-3 | 2mg/1 | TABLET | ORAL | 71335-1719 | 71335-1719-03 | Oral estradiol |
| Estradiol | Estradiol | 71335-1719-4 | 2mg/1 | TABLET | ORAL | 71335-1719 | 71335-1719-04 | Oral estradiol |
| Estradiol | Estradiol | 71335-1719-5 | 2mg/1 | TABLET | ORAL | 71335-1719 | 71335-1719-05 | Oral estradiol |
| Estradiol | Estradiol | 71335-1973-1 | .5mg/1 | TABLET | ORAL | 71335-1973 | 71335-1973-01 | Oral estradiol |
| Estradiol | Estradiol | 71335-1973-2 | .5mg/1 | TABLET | ORAL | 71335-1973 | 71335-1973-02 | Oral estradiol |
| Estradiol | Estradiol | 71335-1973-3 | .5mg/1 | TABLET | ORAL | 71335-1973 | 71335-1973-03 | Oral estradiol |
| Estradiol | Estradiol | 71335-1973-4 | .5mg/1 | TABLET | ORAL | 71335-1973 | 71335-1973-04 | Oral estradiol |
| Estradiol | Estradiol | 71335-2387-1 | 1mg/1 | TABLET | ORAL | 71335-2387 | 71335-2387-01 | Oral estradiol |
| Estradiol | Estradiol | 71335-2387-2 | 1mg/1 | TABLET | ORAL | 71335-2387 | 71335-2387-02 | Oral estradiol |
| Estradiol | Estradiol | 71335-2387-3 | 1mg/1 | TABLET | ORAL | 71335-2387 | 71335-2387-03 | Oral estradiol |
| Estradiol | Estradiol | 71335-2387-4 | 1mg/1 | TABLET | ORAL | 71335-2387 | 71335-2387-04 | Oral estradiol |
| Estradiol | Estradiol | 71335-2387-5 | 1mg/1 | TABLET | ORAL | 71335-2387 | 71335-2387-05 | Oral estradiol |
| Estradiol | Estradiol | 71335-2387-6 | 1mg/1 | TABLET | ORAL | 71335-2387 | 71335-2387-06 | Oral estradiol |
| Estradiol | Estradiol | 71335-2387-7 | 1mg/1 | TABLET | ORAL | 71335-2387 | 71335-2387-07 | Oral estradiol |
| Estradiol | Estradiol | 71335-2477-1 | 2mg/1 | TABLET | ORAL | 71335-2477 | 71335-2477-01 | Oral estradiol |
| Estradiol | Estradiol | 71335-2477-2 | 2mg/1 | TABLET | ORAL | 71335-2477 | 71335-2477-02 | Oral estradiol |
| Estradiol | Estradiol | 71335-2477-3 | 2mg/1 | TABLET | ORAL | 71335-2477 | 71335-2477-03 | Oral estradiol |
| Estradiol | Estradiol | 71335-2477-4 | 2mg/1 | TABLET | ORAL | 71335-2477 | 71335-2477-04 | Oral estradiol |
| Estradiol | Estradiol | 71335-2477-5 | 2mg/1 | TABLET | ORAL | 71335-2477 | 71335-2477-05 | Oral estradiol |
| ESTRADIOL | estradiol | 72162-2290-3 | .25mg/.25g | GEL | TOPICAL | 72162-2290 | 72162-2290-03 | Transdermal estrogen |
| ESTRADIOL | estradiol | 72162-2291-3 | .5mg/.5g | GEL | TOPICAL | 72162-2291 | 72162-2291-03 | Transdermal estrogen |
| ESTRADIOL | estradiol | 72162-2292-3 | .75mg/.75g | GEL | TOPICAL | 72162-2292 | 72162-2292-03 | Transdermal estrogen |
| ESTRADIOL | estradiol | 72162-2293-3 | 1mg/g | GEL | TOPICAL | 72162-2293 | 72162-2293-03 | Transdermal estrogen |
| Estradiol | Estradiol | 72189-502-60 | 1mg/1 | TABLET | ORAL | 72189-502 | 72189-0502-60 | Oral estradiol |
| Estradiol | Estradiol | 72189-502-90 | 1mg/1 | TABLET | ORAL | 72189-502 | 72189-0502-90 | Oral estradiol |
| Estradiol | Estradiol | 72189-508-90 | 2mg/1 | TABLET | ORAL | 72189-508 | 72189-0508-90 | Oral estradiol |
| Estradiol | Estradiol | 72603-241-30 | .25mg/.25g | GEL | TOPICAL | 72603-241 | 72603-0241-30 | Transdermal estrogen |
| Estradiol | Estradiol | 72603-242-30 | .5mg/.5g | GEL | TOPICAL | 72603-242 | 72603-0242-30 | Transdermal estrogen |
| Estradiol | Estradiol | 72603-243-30 | .75mg/.75g | GEL | TOPICAL | 72603-243 | 72603-0243-30 | Transdermal estrogen |
| Estradiol | Estradiol | 72603-244-30 | 1mg/g | GEL | TOPICAL | 72603-244 | 72603-0244-30 | Transdermal estrogen |
| Estradiol | Estradiol | 72603-245-30 | 1.25mg/1.25g | GEL | TOPICAL | 72603-245 | 72603-0245-30 | Transdermal estrogen |
| Estradiol | Estradiol | 72603-273-01 | .5mg/1 | TABLET | ORAL | 72603-273 | 72603-0273-01 | Oral estradiol |
| Estradiol | Estradiol | 72603-274-01 | 1mg/1 | TABLET | ORAL | 72603-274 | 72603-0274-01 | Oral estradiol |
| Estradiol | Estradiol | 72603-274-02 | 1mg/1 | TABLET | ORAL | 72603-274 | 72603-0274-02 | Oral estradiol |
| Estradiol | Estradiol | 72603-275-01 | 2mg/1 | TABLET | ORAL | 72603-275 | 72603-0275-01 | Oral estradiol |
| Estradiol | Estradiol | 72603-275-02 | 2mg/1 | TABLET | ORAL | 72603-275 | 72603-0275-02 | Oral estradiol |
| Estradiol | Estradiol | 72789-299-30 | 1mg/1 | TABLET | ORAL | 72789-299 | 72789-0299-30 | Oral estradiol |
| Estradiol | Estradiol | 72789-299-90 | 1mg/1 | TABLET | ORAL | 72789-299 | 72789-0299-90 | Oral estradiol |
| Estradiol | Estradiol | 72789-360-90 | 2mg/1 | TABLET | ORAL | 72789-360 | 72789-0360-90 | Oral estradiol |
| Estradiol | Estradiol | 72789-489-30 | .5mg/1 | TABLET | ORAL | 72789-489 | 72789-0489-30 | Oral estradiol |
| Estradiol | ESTRADIOL | 73473-308-50 | .75mg/1.25g | GEL | TOPICAL | 73473-308 | 73473-0308-50 | Transdermal estrogen |
| ESTRADIOL | ESTRADIOL | 76420-021-30 | .5mg/1 | TABLET | ORAL | 76420-021 | 76420-0021-30 | Oral estradiol |
| ESTRADIOL | ESTRADIOL | 76420-021-90 | .5mg/1 | TABLET | ORAL | 76420-021 | 76420-0021-90 | Oral estradiol |
| ESTRADIOL | ESTRADIOL | 76420-021-10 | .5mg/1 | TABLET | ORAL | 76420-021 | 76420-0021-10 | Oral estradiol |
| ESTRADIOL | ESTRADIOL | 76420-021-30 | .5mg/1 | TABLET | ORAL | 76420-021 | 76420-0021-30 | Oral estradiol |
| ESTRADIOL | ESTRADIOL | 76420-021-90 | .5mg/1 | TABLET | ORAL | 76420-021 | 76420-0021-90 | Oral estradiol |
| ESTRADIOL | ESTRADIOL | 76420-021-10 | .5mg/1 | TABLET | ORAL | 76420-021 | 76420-0021-10 | Oral estradiol |
| ESTRADIOL | ESTRADIOL | 76420-022-30 | 1mg/1 | TABLET | ORAL | 76420-022 | 76420-0022-30 | Oral estradiol |
| ESTRADIOL | ESTRADIOL | 76420-022-90 | 1mg/1 | TABLET | ORAL | 76420-022 | 76420-0022-90 | Oral estradiol |
| ESTRADIOL | ESTRADIOL | 76420-022-10 | 1mg/1 | TABLET | ORAL | 76420-022 | 76420-0022-10 | Oral estradiol |
| ESTRADIOL | ESTRADIOL | 76420-022-30 | 1mg/1 | TABLET | ORAL | 76420-022 | 76420-0022-30 | Oral estradiol |
| ESTRADIOL | ESTRADIOL | 76420-022-90 | 1mg/1 | TABLET | ORAL | 76420-022 | 76420-0022-90 | Oral estradiol |
| ESTRADIOL | ESTRADIOL | 76420-022-10 | 1mg/1 | TABLET | ORAL | 76420-022 | 76420-0022-10 | Oral estradiol |
| ESTRADIOL | ESTRADIOL | 76420-023-30 | 2mg/1 | TABLET | ORAL | 76420-023 | 76420-0023-30 | Oral estradiol |
| ESTRADIOL | ESTRADIOL | 76420-023-90 | 2mg/1 | TABLET | ORAL | 76420-023 | 76420-0023-90 | Oral estradiol |
| ESTRADIOL | ESTRADIOL | 76420-023-10 | 2mg/1 | TABLET | ORAL | 76420-023 | 76420-0023-10 | Oral estradiol |
| ESTRADIOL | ESTRADIOL | 76420-023-30 | 2mg/1 | TABLET | ORAL | 76420-023 | 76420-0023-30 | Oral estradiol |
| ESTRADIOL | ESTRADIOL | 76420-023-90 | 2mg/1 | TABLET | ORAL | 76420-023 | 76420-0023-90 | Oral estradiol |
| ESTRADIOL | ESTRADIOL | 76420-023-10 | 2mg/1 | TABLET | ORAL | 76420-023 | 76420-0023-10 | Oral estradiol |
| Estradiol | Estradiol | 82868-026-30 | 2mg/1 | TABLET | ORAL | 82868-026 | 82868-0026-30 | Oral estradiol |
| Estradiol | Estradiol | 82868-038-30 | 1mg/1 | TABLET | ORAL | 82868-038 | 82868-0038-30 | Oral estradiol |
| Estradiol | Estradiol | 82868-038-90 | 1mg/1 | TABLET | ORAL | 82868-038 | 82868-0038-90 | Oral estradiol |
| Estradiol / Norethindrone Acetate | Estradiol and Norethindrone Acetate | 51991-474-28 | 1mg/1, .5 mg/1 | TABLET, FILM COATED | ORAL | 51991-474 | 51991-0474-28 | Combined estrogen–progestogen therapy |
| Estradiol / Norethindrone Acetate | Estradiol and Norethindrone Acetate | 51991-623-28 | .5mg/1, .1 mg/1 | TABLET, FILM COATED | ORAL | 51991-623 | 51991-0623-28 | Combined estrogen–progestogen therapy |
| ESTRADIOL AND NORETHINDRONE ACETATE | estradiol and norethindrone acetate | 0378-7294-53 | .5mg/1, .1 mg/1 | TABLET | ORAL | 0378-7294 | 00378-7294-53 | Combined estrogen–progestogen therapy |
| ESTRADIOL AND NORETHINDRONE ACETATE | estradiol and norethindrone acetate | 0378-7295-53 | 1mg/1, .5 mg/1 | TABLET | ORAL | 0378-7295 | 00378-7295-53 | Combined estrogen–progestogen therapy |
| Estradiol and Norethindrone Acetate | Estradiol and Norethindrone Acetate | 50742-657-28 | 1mg/1, .5 mg/1 | TABLET | ORAL | 50742-657 | 50742-0657-28 | Combined estrogen–progestogen therapy |
| Estradiol and Norethindrone Acetate | Estradiol and Norethindrone Acetate | 50742-657-68 | 1mg/1, .5 mg/1 | TABLET | ORAL | 50742-657 | 50742-0657-68 | Combined estrogen–progestogen therapy |
| Estradiol and Norethindrone Acetate | Estradiol and Norethindrone Acetate | 50742-657-84 | 1mg/1, .5 mg/1 | TABLET | ORAL | 50742-657 | 50742-0657-84 | Combined estrogen–progestogen therapy |
| Estradiol and Norethindrone Acetate | Estradiol and Norethindrone Acetate | 50742-658-28 | .5mg/1, .1 mg/1 | TABLET | ORAL | 50742-658 | 50742-0658-28 | Combined estrogen–progestogen therapy |
| Estradiol and Norethindrone Acetate | Estradiol and Norethindrone Acetate | 50742-658-68 | .5mg/1, .1 mg/1 | TABLET | ORAL | 50742-658 | 50742-0658-68 | Combined estrogen–progestogen therapy |
| Estradiol and Norethindrone Acetate | Estradiol and Norethindrone Acetate | 50742-658-84 | .5mg/1, .1 mg/1 | TABLET | ORAL | 50742-658 | 50742-0658-84 | Combined estrogen–progestogen therapy |
| ESTRADIOL AND NORETHINDRONE ACETATE | estradiol and norethindrone acetate | 79929-012-07 | 1mg/1, .5 mg/1 | TABLET | ORAL | 79929-012 | 79929-0012-07 | Combined estrogen–progestogen therapy |
| Estradiol and Progesterone | Estradiol and Progesterone | 69238-1690-3 | 1mg/1, 100 mg/1 | CAPSULE | ORAL | 69238-1690 | 69238-1690-03 | Combined estrogen–progestogen therapy |
| Estradiol Gel | Estradiol | 21922-086-52 | .25mg/.25g | GEL | TOPICAL | 21922-086 | 21922-0086-52 | Transdermal estrogen |
| Estradiol Gel | Estradiol | 21922-087-52 | .5mg/.5g | GEL | TOPICAL | 21922-087 | 21922-0087-52 | Transdermal estrogen |
| Estradiol Gel | Estradiol | 21922-088-52 | .75mg/.75g | GEL | TOPICAL | 21922-088 | 21922-0088-52 | Transdermal estrogen |
| Estradiol Gel | Estradiol | 21922-089-52 | 1mg/g | GEL | TOPICAL | 21922-089 | 21922-0089-52 | Transdermal estrogen |
| Estradiol Gel | Estradiol | 21922-090-52 | 1.25mg/1.25g | GEL | TOPICAL | 21922-090 | 21922-0090-52 | Transdermal estrogen |
| Estradiol Transdermal System | Estradiol | 0781-7104-54 | .1mg/d | PATCH | TRANSDERMAL | 0781-7104 | 00781-7104-54 | Transdermal estrogen |
| Estradiol Transdermal System | Estradiol | 0781-7119-54 | .025mg/d | PATCH | TRANSDERMAL | 0781-7119 | 00781-7119-54 | Transdermal estrogen |
| Estradiol Transdermal System | Estradiol | 0781-7122-54 | .0375mg/d | PATCH | TRANSDERMAL | 0781-7122 | 00781-7122-54 | Transdermal estrogen |
| Estradiol Transdermal System | Estradiol | 0781-7133-54 | .05mg/d | PATCH | TRANSDERMAL | 0781-7133 | 00781-7133-54 | Transdermal estrogen |
| Estradiol Transdermal System | Estradiol | 0781-7134-54 | .06mg/d | PATCH | TRANSDERMAL | 0781-7134 | 00781-7134-54 | Transdermal estrogen |
| Estradiol Transdermal System | Estradiol | 0781-7136-54 | .075mg/d | PATCH | TRANSDERMAL | 0781-7136 | 00781-7136-54 | Transdermal estrogen |
| Estradiol Vaginal | Estradiol | 42291-426-42 | .1mg/g | CREAM | VAGINAL | 42291-426 | 42291-0426-42 | Vaginal estrogen |
| Estradiol Vaginal | Estradiol | 45802-097-35 | .1mg/g | CREAM | VAGINAL | 45802-097 | 45802-0097-35 | Vaginal estrogen |
| Estradiol Vaginal | estradiol | 50090-6811-0 | .1mg/g | CREAM | VAGINAL | 50090-6811 | 50090-6811-00 | Vaginal estrogen |
| Estradiol Vaginal | Estradiol | 50090-7266-0 | .1mg/g | CREAM | VAGINAL | 50090-7266 | 50090-7266-00 | Vaginal estrogen |
| Estradiol Vaginal | Estradiol | 63629-8654-1 | .1mg/g | CREAM | VAGINAL | 63629-8654 | 63629-8654-01 | Vaginal estrogen |
| Estradiol Vaginal | Estradiol | 68071-3796-4 | .1mg/g | CREAM | VAGINAL | 68071-3796 | 68071-3796-04 | Vaginal estrogen |
| Estradiol Vaginal | Estradiol | 72162-1389-2 | .1mg/g | CREAM | VAGINAL | 72162-1389 | 72162-1389-02 | Vaginal estrogen |
| Estradiol Vaginal Cream | estradiol | 66993-002-10 | .1mg/g | CREAM | VAGINAL | 66993-002 | 66993-0002-10 | Vaginal estrogen |
| ESTRADIOL VAGINAL INSERTS | Estradiol | 59651-439-08 | 10ug/1 | TABLET, FILM COATED | VAGINAL | 59651-439 | 59651-0439-08 | Vaginal estrogen |
| ESTRADIOL VAGINAL INSERTS | Estradiol | 59651-439-18 | 10ug/1 | TABLET, FILM COATED | VAGINAL | 59651-439 | 59651-0439-18 | Vaginal estrogen |
| ESTRADIOL VAGINAL INSERTS | estradiol | 68462-711-71 | 10ug/1 | TABLET, FILM COATED | VAGINAL | 68462-711 | 68462-0711-71 | Vaginal estrogen |
| ESTRADIOL VAGINAL INSERTS | estradiol | 68462-711-88 | 10ug/1 | TABLET, FILM COATED | VAGINAL | 68462-711 | 68462-0711-88 | Vaginal estrogen |
| Estradiol Valerate | Estradiol Valerate | 0143-9289-01 | 10mg/mL | INJECTION | INTRAMUSCULAR | 0143-9289 | 00143-9289-01 | Intramuscular estrogen |
| Estradiol Valerate | Estradiol Valerate | 0143-9290-01 | 20mg/mL | INJECTION | INTRAMUSCULAR | 0143-9290 | 00143-9290-01 | Intramuscular estrogen |
| Estradiol Valerate | Estradiol Valerate | 0143-9291-01 | 40mg/mL | INJECTION | INTRAMUSCULAR | 0143-9291 | 00143-9291-01 | Intramuscular estrogen |
| ESTRADIOL VALERATE | ESTRADIOL VALERATE | 0517-0420-01 | 20mg/mL | INJECTION, SOLUTION | INTRAMUSCULAR | 0517-0420 | 00517-0420-01 | Intramuscular estrogen |
| ESTRADIOL VALERATE | ESTRADIOL VALERATE | 0517-0440-01 | 40mg/mL | INJECTION, SOLUTION | INTRAMUSCULAR | 0517-0440 | 00517-0440-01 | Intramuscular estrogen |
| Estradiol valerate | Estradiol valerate | 70700-273-22 | 10mg/mL | INJECTION | INTRAMUSCULAR | 70700-273 | 70700-0273-22 | Intramuscular estrogen |
| Estradiol valerate | Estradiol valerate | 70700-274-22 | 20mg/mL | INJECTION | INTRAMUSCULAR | 70700-274 | 70700-0274-22 | Intramuscular estrogen |
| Estradiol valerate | Estradiol valerate | 70700-275-22 | 40mg/mL | INJECTION | INTRAMUSCULAR | 70700-275 | 70700-0275-22 | Intramuscular estrogen |
| Estring | estradiol | 0013-1042-01 | 2mg/1 | SYSTEM | VAGINAL | 0013-1042 | 00013-1042-01 | Vaginal estrogen |
| EstroGel | estradiol | 17139-617-40 | .75mg/1.25g | GEL, METERED | TOPICAL | 17139-617 | 17139-0617-40 | Transdermal estrogen |
| Estrogen | Estradiol | 83027-0075-1 | 4[hp_X]/mL | SPRAY | ORAL | 83027-0075 | 83027-0075-01 | Transdermal estrogen |
| Etyqa | ESTRADIOL AND NORETHINDRONE ACETATE | 59651-424-28 | 1mg/1, .5 mg/1 | TABLET, FILM COATED | ORAL | 59651-424 | 59651-0424-28 | Combined estrogen–progestogen therapy |
| Etyqa | ESTRADIOL AND NORETHINDRONE ACETATE | 59651-424-87 | 1mg/1, .5 mg/1 | TABLET, FILM COATED | ORAL | 59651-424 | 59651-0424-87 | Combined estrogen–progestogen therapy |
| Etyqa | ESTRADIOL AND NORETHINDRONE ACETATE | 59651-425-28 | .5mg/1, .1 mg/1 | TABLET, FILM COATED | ORAL | 59651-425 | 59651-0425-28 | Combined estrogen–progestogen therapy |
| Etyqa | ESTRADIOL AND NORETHINDRONE ACETATE | 59651-425-87 | .5mg/1, .1 mg/1 | TABLET, FILM COATED | ORAL | 59651-425 | 59651-0425-87 | Combined estrogen–progestogen therapy |
| Evamist | Estradiol | 0574-2067-00 | 1.53mg/1 | SPRAY | TRANSDERMAL | 0574-2067 | 00574-2067-00 | Transdermal estrogen |
| Evamist | Estradiol | 0574-2067-27 | 1.53mg/1 | SPRAY | TRANSDERMAL | 0574-2067 | 00574-2067-27 | Transdermal estrogen |
| Femring | estradiol acetate | 72495-201-05 | .05mg/d | RING | VAGINAL | 72495-201 | 72495-0201-05 | Vaginal estrogen |
| Femring | estradiol acetate | 72495-202-10 | .1mg/d | RING | VAGINAL | 72495-202 | 72495-0202-10 | Vaginal estrogen |
| IMVEXXY | estradiol | 50261-110-08 | 10ug/1 | INSERT | VAGINAL | 50261-110 | 50261-0110-08 | Vaginal estrogen |
| IMVEXXY | estradiol | 50261-110-18 | 10ug/1 | INSERT | VAGINAL | 50261-110 | 50261-0110-18 | Vaginal estrogen |
| Imvexxy | estradiol | 68308-747-00 | 4ug/1 | INSERT | VAGINAL | 68308-747 | 68308-0747-00 | Vaginal estrogen |
| Imvexxy | estradiol | 68308-747-08 | 4ug/1 | INSERT | VAGINAL | 68308-747 | 68308-0747-08 | Vaginal estrogen |
| Imvexxy | estradiol | 68308-747-18 | 4ug/1 | INSERT | VAGINAL | 68308-747 | 68308-0747-18 | Vaginal estrogen |
| Imvexxy | estradiol | 68308-748-00 | 10ug/1 | INSERT | VAGINAL | 68308-748 | 68308-0748-00 | Vaginal estrogen |
| Imvexxy | estradiol | 68308-748-08 | 10ug/1 | INSERT | VAGINAL | 68308-748 | 68308-0748-08 | Vaginal estrogen |
| Imvexxy | estradiol | 68308-748-18 | 10ug/1 | INSERT | VAGINAL | 68308-748 | 68308-0748-18 | Vaginal estrogen |
| Lopreeza | estradiol/norethindrone acetate | 69238-1251-8 | 1mg/1, .5 mg/1 | TABLET, FILM COATED | ORAL | 69238-1251 | 69238-1251-08 | Combined estrogen–progestogen therapy |
| Lopreeza | estradiol/norethindrone acetate | 69238-1252-8 | .5mg/1, .1 mg/1 | TABLET, FILM COATED | ORAL | 69238-1252 | 69238-1252-08 | Combined estrogen–progestogen therapy |
| LOPREEZA | estradiol/norethindrone acetate | 69238-1609-6 | .5mg/1, .1 mg/1 | TABLET, FILM COATED | ORAL | 69238-1609 | 69238-1609-06 | Combined estrogen–progestogen therapy |
| LOPREEZA | estradiol/norethindrone acetate | 69238-1610-6 | 1mg/1, .5 mg/1 | TABLET, FILM COATED | ORAL | 69238-1610 | 69238-1610-06 | Combined estrogen–progestogen therapy |
| LYLLANA | Estradiol | 65162-126-08 | .025mg/d | PATCH, EXTENDED RELEASE | TRANSDERMAL | 65162-126 | 65162-0126-08 | Transdermal estrogen |
| LYLLANA | Estradiol | 65162-148-08 | .0375mg/d | PATCH, EXTENDED RELEASE | TRANSDERMAL | 65162-148 | 65162-0148-08 | Transdermal estrogen |
| LYLLANA | Estradiol | 65162-149-08 | .05mg/d | PATCH, EXTENDED RELEASE | TRANSDERMAL | 65162-149 | 65162-0149-08 | Transdermal estrogen |
| LYLLANA | Estradiol | 65162-150-08 | .075mg/d | PATCH, EXTENDED RELEASE | TRANSDERMAL | 65162-150 | 65162-0150-08 | Transdermal estrogen |
| LYLLANA | Estradiol | 65162-228-08 | .1mg/d | PATCH, EXTENDED RELEASE | TRANSDERMAL | 65162-228 | 65162-0228-08 | Transdermal estrogen |
| Menostar | estradiol | 50419-455-04 | 14ug/d | PATCH | TRANSDERMAL | 50419-455 | 50419-0455-04 | Transdermal estrogen |
| Mimvey | Estradiol and Norethindrone Acetate | 0093-5455-28 | 1mg/1, .5 mg/1 | TABLET, FILM COATED | ORAL | 0093-5455 | 00093-5455-28 | Combined estrogen–progestogen therapy |
| Mimvey | Estradiol and Norethindrone Acetate | 0093-5455-42 | 1mg/1, .5 mg/1 | TABLET, FILM COATED | ORAL | 0093-5455 | 00093-5455-42 | Combined estrogen–progestogen therapy |
| Minivelle | estradiol | 68968-6610-8 | .1mg/d | FILM, EXTENDED RELEASE | TRANSDERMAL | 68968-6610 | 68968-6610-08 | Transdermal estrogen |
| Minivelle | estradiol | 68968-6625-8 | .025mg/d | FILM, EXTENDED RELEASE | TRANSDERMAL | 68968-6625 | 68968-6625-08 | Transdermal estrogen |
| Minivelle | estradiol | 68968-6637-8 | .0375mg/d | FILM, EXTENDED RELEASE | TRANSDERMAL | 68968-6637 | 68968-6637-08 | Transdermal estrogen |
| Minivelle | estradiol | 68968-6650-8 | .05mg/d | FILM, EXTENDED RELEASE | TRANSDERMAL | 68968-6650 | 68968-6650-08 | Transdermal estrogen |
| Minivelle | estradiol | 68968-6675-8 | .075mg/d | FILM, EXTENDED RELEASE | TRANSDERMAL | 68968-6675 | 68968-6675-08 | Transdermal estrogen |
| Vagifem | estradiol | 0169-5176-03 | 10ug/1 | INSERT | VAGINAL | 0169-5176 | 00169-5176-03 | Vaginal estrogen |
| Vagifem | estradiol | 0169-5176-04 | 10ug/1 | INSERT | VAGINAL | 0169-5176 | 00169-5176-04 | Vaginal estrogen |
| Vagifem | estradiol | 0169-5176-99 | 10ug/1 | INSERT | VAGINAL | 0169-5176 | 00169-5176-99 | Vaginal estrogen |
| Vivelle-Dot | estradiol | 0078-0343-42 | .0375mg/d | PATCH, EXTENDED RELEASE | TRANSDERMAL | 0078-0343 | 00078-0343-42 | Transdermal estrogen |
| Vivelle-Dot | estradiol | 0078-0344-42 | .05mg/d | PATCH, EXTENDED RELEASE | TRANSDERMAL | 0078-0344 | 00078-0344-42 | Transdermal estrogen |
| Vivelle-Dot | estradiol | 0078-0345-42 | .075mg/d | PATCH, EXTENDED RELEASE | TRANSDERMAL | 0078-0345 | 00078-0345-42 | Transdermal estrogen |
| Vivelle-Dot | estradiol | 0078-0346-42 | .1mg/d | PATCH, EXTENDED RELEASE | TRANSDERMAL | 0078-0346 | 00078-0346-42 | Transdermal estrogen |
| Vivelle-Dot | estradiol | 0078-0365-42 | .025mg/d | PATCH, EXTENDED RELEASE | TRANSDERMAL | 0078-0365 | 00078-0365-42 | Transdermal estrogen |
| Vivelle-Dot | estradiol | 66758-145-83 | .025mg/d | PATCH, EXTENDED RELEASE | TRANSDERMAL | 66758-145 | 66758-0145-83 | Transdermal estrogen |
| Vivelle-Dot | estradiol | 66758-146-83 | .0375mg/d | PATCH, EXTENDED RELEASE | TRANSDERMAL | 66758-146 | 66758-0146-83 | Transdermal estrogen |
| Vivelle-Dot | estradiol | 66758-147-83 | .05mg/d | PATCH, EXTENDED RELEASE | TRANSDERMAL | 66758-147 | 66758-0147-83 | Transdermal estrogen |
| Vivelle-Dot | estradiol | 66758-148-83 | .075mg/d | PATCH, EXTENDED RELEASE | TRANSDERMAL | 66758-148 | 66758-0148-83 | Transdermal estrogen |
| Vivelle-Dot | estradiol | 66758-149-83 | .1mg/d | PATCH, EXTENDED RELEASE | TRANSDERMAL | 66758-149 | 66758-0149-83 | Transdermal estrogen |
| Yuvafem | Estradiol | 42291-962-08 | 10ug/1 | TABLET | VAGINAL | 42291-962 | 42291-0962-08 | Vaginal estrogen |
| Yuvafem | Estradiol | 42291-962-18 | 10ug/1 | TABLET | VAGINAL | 42291-962 | 42291-0962-18 | Vaginal estrogen |
| YUVAFEM | Estradiol | 65162-226-21 | 10ug/1 | TABLET | VAGINAL | 65162-226 | 65162-0226-21 | Vaginal estrogen |
| YUVAFEM | Estradiol | 65162-226-23 | 10ug/1 | TABLET | VAGINAL | 65162-226 | 65162-0226-23 | Vaginal estrogen |
| Esterified Estrogens and Methyltestosterone | Esterified Estrogens and Methyltestosterone | 53746-077-01 | .625mg/1, 1.25 mg/1 | TABLET | ORAL | 53746-077 | 53746-0077-01 | Oral esterified estrogens |
| Esterified Estrogens and Methyltestosterone | Esterified Estrogens and Methyltestosterone | 53746-078-01 | 1.25mg/1, 2.5 mg/1 | TABLET | ORAL | 53746-078 | 53746-0078-01 | Oral esterified estrogens |
| ESTERIFIED ESTROGENS AND METHYLTESTOSTERONE | ESTERIFIED ESTROGENS, METHYLTESTOSTERONE | 58657-407-01 | .625mg/1, 1.25 mg/1 | TABLET | ORAL | 58657-407 | 58657-0407-01 | Oral esterified estrogens |
| ESTERIFIED ESTROGENS AND METHYLTESTOSTERONE | ESTERIFIED ESTROGENS, METHYLTESTOSTERONE | 58657-408-01 | 1.25mg/1, 2.5 mg/1 | TABLET | ORAL | 58657-408 | 58657-0408-01 | Oral esterified estrogens |
| Esterified Estrogens and Methyltestosterone | Esterified Estrogens and Methyltestosterone | 62559-149-01 | 1.25mg/1, 2.5 mg/1 | TABLET | ORAL | 62559-149 | 62559-0149-01 | Oral esterified estrogens |
| Esterified Estrogens and Methyltestosterone | Esterified Estrogens and Methyltestosterone | 62559-150-01 | .625mg/1, 1.25 mg/1 | TABLET | ORAL | 62559-150 | 62559-0150-01 | Oral esterified estrogens |
| Esterified Estrogens and Methyltestosterone | Esterified Estrogens and Methyltestosterone | 63629-1979-1 | 1.25mg/1, 2.5 mg/1 | TABLET | ORAL | 63629-1979 | 63629-1979-01 | Oral esterified estrogens |
| Esterified Estrogens and Methyltestosterone | Esterified Estrogens and Methyltestosterone | 63629-1980-1 | .625mg/1, 1.25 mg/1 | TABLET | ORAL | 63629-1980 | 63629-1980-01 | Oral esterified estrogens |
| Esterified Estrogens and Methyltestosterone | Esterified Estrogens and Methyltestosterone | 63629-9177-1 | 1.25mg/1, 2.5 mg/1 | TABLET | ORAL | 63629-9177 | 63629-9177-01 | Oral esterified estrogens |
| Esterified Estrogens and Methyltestosterone | Esterified Estrogens and Methyltestosterone | 63629-9178-1 | .625mg/1, 1.25 mg/1 | TABLET | ORAL | 63629-9178 | 63629-9178-01 | Oral esterified estrogens |
| ESTERIFIED ESTROGENS AND METHYLTESTOSTERONE | ESTERIFIED ESTROGENS, METHYLTESTOSTERONE | 69315-283-01 | .625mg/1, 1.25 mg/1 | TABLET | ORAL | 69315-283 | 69315-0283-01 | Oral esterified estrogens |
| ESTERIFIED ESTROGENS AND METHYLTESTOSTERONE | ESTERIFIED ESTROGENS, METHYLTESTOSTERONE | 69315-284-01 | 1.25mg/1, 2.5 mg/1 | TABLET | ORAL | 69315-284 | 69315-0284-01 | Oral esterified estrogens |
| Esterified Estrogens and Methyltestosterone | Esterified Estrogens and Methyltestosterone | 72162-1747-1 | 1.25mg/1, 2.5 mg/1 | TABLET | ORAL | 72162-1747 | 72162-1747-01 | Oral esterified estrogens |
| Esterified Estrogens and Methyltestosterone | Esterified Estrogens and Methyltestosterone | 72162-1748-1 | .625mg/1, 1.25 mg/1 | TABLET | ORAL | 72162-1748 | 72162-1748-01 | Oral esterified estrogens |
| ESTERIFIED ESTROGENS AND METHYLTESTOSTERONE | ESTERIFIED ESTROGENS, METHYLTESTOSTERONE | 72162-2393-1 | 1.25mg/1, 2.5 mg/1 | TABLET | ORAL | 72162-2393 | 72162-2393-01 | Oral esterified estrogens |
| Esterified Estrogens and Methyltestosterone | Esterified Estrogens, Methyltestosterone | 84346-450-01 | .625mg/1, 1.25 mg/1 | TABLET | ORAL | 84346-450 | 84346-0450-01 | Oral esterified estrogens |
| ESTERIFIED ESTROGENS AND METHYLTESTOSTERONE FULL STRENGTH | ESTERIFIED ESTROGENS, METHYLTESTOSTERONE | 58657-419-01 | 1.25mg/1, 2.5 mg/1 | TABLET | ORAL | 58657-419 | 58657-0419-01 | Oral esterified estrogens |
| ESTERIFIED ESTROGENS AND METHYLTESTOSTERONE HALF STRENGTH | ESTERIFIED ESTROGENS, METHYLTESTOSTERONE | 58657-418-01 | .625mg/1, 1.25 mg/1 | TABLET | ORAL | 58657-418 | 58657-0418-01 | Oral esterified estrogens |
| ESTRATEST F.S. | ESTERIFIED ESTROGENS, METHYLTESTOSTERONE | 58657-404-01 | 1.25mg/1, 2.5 mg/1 | TABLET | ORAL | 58657-404 | 58657-0404-01 | Oral esterified estrogens |
| ESTRATEST F.S. | ESTERIFIED ESTROGENS, METHYLTESTOSTERONE | 72162-2392-1 | 1.25mg/1, 2.5 mg/1 | TABLET | ORAL | 72162-2392 | 72162-2392-01 | Oral esterified estrogens |
| Estratest FS | Esterified Estrogens and Methyltestosterone | 58657-417-01 | 1.25mg/1, 2.5 mg/1 | TABLET | ORAL | 58657-417 | 58657-0417-01 | Oral esterified estrogens |
| ESTRATEST H.S. | ESTERIFIED ESTROGENS, METHYLTESTOSTERONE | 58657-403-01 | .625mg/1, 1.25 mg/1 | TABLET | ORAL | 58657-403 | 58657-0403-01 | Oral esterified estrogens |
| Estratest HS | Esterified Estrogens and Methyltestosterone | 58657-416-01 | .625mg/1, 1.25 mg/1 | TABLET | ORAL | 58657-416 | 58657-0416-01 | Oral esterified estrogens |
| MENEST | esterified estrogens | 61570-072-01 | .3mg/1 | TABLET, FILM COATED | ORAL | 61570-072 | 61570-0072-01 | Oral esterified estrogens |
| MENEST | esterified estrogens | 61570-073-01 | .625mg/1 | TABLET, FILM COATED | ORAL | 61570-073 | 61570-0073-01 | Oral esterified estrogens |
| MENEST | esterified estrogens | 61570-074-01 | 1.25mg/1 | TABLET, FILM COATED | ORAL | 61570-074 | 61570-0074-01 | Oral esterified estrogens |
| MENEST | esterified estrogens | 61570-075-50 | 2.5mg/1 | TABLET, FILM COATED | ORAL | 61570-075 | 61570-0075-50 | Oral esterified estrogens |
| Premarin | conjugated estrogens | 0046-0872-04 | .625mg/g | CREAM | VAGINAL | 0046-0872 | 00046-0872-04 | Vaginal estrogen |
| Premarin | conjugated estrogens | 0046-0872-21 | .625mg/g | CREAM | VAGINAL | 0046-0872 | 00046-0872-21 | Vaginal estrogen |
| Premarin | estrogens, conjugated | 0046-1100-52 | .3mg/1 | TABLET, FILM COATED | ORAL | 0046-1100 | 00046-1100-52 | Oral conjugated estrogens |
| Premarin | estrogens, conjugated | 0046-1100-81 | .3mg/1 | TABLET, FILM COATED | ORAL | 0046-1100 | 00046-1100-81 | Oral conjugated estrogens |
| Premarin | estrogens, conjugated | 0046-1100-91 | .3mg/1 | TABLET, FILM COATED | ORAL | 0046-1100 | 00046-1100-91 | Oral conjugated estrogens |
| Premarin | estrogens, conjugated | 0046-1101-81 | .45mg/1 | TABLET, FILM COATED | ORAL | 0046-1101 | 00046-1101-81 | Oral conjugated estrogens |
| Premarin | estrogens, conjugated | 0046-1102-52 | .625mg/1 | TABLET, FILM COATED | ORAL | 0046-1102 | 00046-1102-52 | Oral conjugated estrogens |
| Premarin | estrogens, conjugated | 0046-1102-81 | .625mg/1 | TABLET, FILM COATED | ORAL | 0046-1102 | 00046-1102-81 | Oral conjugated estrogens |
| Premarin | estrogens, conjugated | 0046-1102-91 | .625mg/1 | TABLET, FILM COATED | ORAL | 0046-1102 | 00046-1102-91 | Oral conjugated estrogens |
| Premarin | estrogens, conjugated | 0046-1103-81 | .9mg/1 | TABLET, FILM COATED | ORAL | 0046-1103 | 00046-1103-81 | Oral conjugated estrogens |
| Premarin | estrogens, conjugated | 0046-1104-81 | 1.25mg/1 | TABLET, FILM COATED | ORAL | 0046-1104 | 00046-1104-81 | Oral conjugated estrogens |
| Premarin | estrogens, conjugated | 0046-1104-91 | 1.25mg/1 | TABLET, FILM COATED | ORAL | 0046-1104 | 00046-1104-91 | Oral conjugated estrogens |
| Premarin | estrogens, conjugated | 50090-0167-0 | .625mg/1 | TABLET, FILM COATED | ORAL | 50090-0167 | 50090-0167-00 | Oral conjugated estrogens |
| Premarin | estrogens, conjugated | 50090-0167-5 | .625mg/1 | TABLET, FILM COATED | ORAL | 50090-0167 | 50090-0167-05 | Oral conjugated estrogens |
| PREMARIN | estrogens, conjugated | 50090-1853-3 | .3mg/1 | TABLET, FILM COATED | ORAL | 50090-1853 | 50090-1853-03 | Oral conjugated estrogens |
| Prempro | conjugated estrogens and medroxyprogesterone acetate | 0046-1105-11 | .3mg/1, 1.5 mg/1 | TABLET, SUGAR COATED | ORAL | 0046-1105 | 00046-1105-11 | Combined estrogen–progestogen therapy |
| Prempro | conjugated estrogens and medroxyprogesterone acetate | 0046-1106-11 | .45mg/1, 1.5 mg/1 | TABLET, SUGAR COATED | ORAL | 0046-1106 | 00046-1106-11 | Combined estrogen–progestogen therapy |
| Prempro | conjugated estrogens and medroxyprogesterone acetate | 0046-1107-11 | .625mg/1, 2.5 mg/1 | TABLET, SUGAR COATED | ORAL | 0046-1107 | 00046-1107-11 | Combined estrogen–progestogen therapy |
| Prempro | conjugated estrogens and medroxyprogesterone acetate | 0046-1108-11 | .625mg/1, 5 mg/1 | TABLET, SUGAR COATED | ORAL | 0046-1108 | 00046-1108-11 | Combined estrogen–progestogen therapy |
